# Traditional Processing Can Enhance the Medicinal Effects of *Polygonatum cyrtonema* by Inducing Significant Chemical Changes in the Functional Components in Its Rhizomes

**DOI:** 10.3390/ph17081074

**Published:** 2024-08-15

**Authors:** Jianjun Shen, Weiting Pu, Qiyan Song, Bihuan Ye, Xiaoxiao Shi, Youwu Chen, Yefei Yu, Haibo Li

**Affiliations:** 1 Zhejiang Academy of Forestry, Hangzhou 310023, China; lky_sjj@126.com (J.S.);; 2 School of Forestry and Biotechnology, Zhejiang Agriculture and Forestry University, Hangzhou 311300, China; 3 Zhejiang Dapanshan National Natural Reserve Administration, Panan 322300, China

**Keywords:** *Polygonatum cyrtonema*, medicinal plant, processing, enhancing the medicinal effects, functional components, chemical changes, unsupervised principal component analysis, hierarchical clustering analysis, orthogonal partial least squares discriminant analysis

## Abstract

The aims of this study were to explore the significant chemical changes in functional components induced by the traditional processing method and evaluate whether this method based on nine cycles of steaming and drying can effectively enhance the medicinal effects of *Polygonatum cyrtonema* rhizome. A global analysis on dynamic changes in secondary metabolites during nine processing cycles was performed, and the significantly differentially accumulated secondary metabolites were initially identified based on the secondary metabolome. Unsupervised principal component analysis (PCA), hierarchical clustering analysis (HCA), and orthogonal partial least squares discriminant analysis (OPLA-DA) on secondary metabolites clearly showed that processing significantly increased the global accumulation of secondary metabolites in processed *P. cyrtonema* rhizomes compared to unprocessed crude rhizomes. The first six processing cycles induced drastic changes in the accumulation of functional components, while the last three did not induce further changes. The accumulations of most functional components were significantly enhanced after the first three cycles and stabilized after six cycles; meanwhile, the first three cycles also led to numerous new components. However, the enhancing effects were unavoidably reversed or weakened under continued processing lasting 6–9 cycles. Furthermore, continued processing also reduced the contents of a small number of original components to undetectable levels. Processing induced some significantly enriched Kyoto Encyclopedia of Genes and Genomes (KEGG) pathways, among which the first three processing cycles enhanced the synthesis of various secondary metabolites and significantly affected the metabolisms of amino acids. In conclusion, this study not only reveals that processing can effectively enhance the medicinal effects, by two main mechanisms including enhancing chemical synthesis and inducing structural transformation of functional components, but also provides theoretical guidance for the optimization of the traditional processing method based on nine cycles of steaming and drying for achieving optimal effects on enhancing the medicinal effects of *P. cyrtonema* rhizome.

## 1. Introduction

Medicinal plants, a rich source of bioactive compounds, provide new chance to discover promising new drugs for their functions on disease prevention and treatment. The extracts from medicinal plants also show the antibacterial effect by the main mechanisms including oxidative stress, inhibition of cell wall synthesis, damage to the cell membrane, and inhibition of nucleic acid and protein synthesis, which is attributed to the abundant secondary metabolites in the extracts, such as phenolic compounds, tannins, terpenoids, flavonoids, and glycosides [1]. Species in the genus *Polygonatum* are perennial herbs of the family Asparagaceae. The species *P. cyrtonema* Hua (“*Duohua Huangjin*” in Chinese), a known medicinal plant, is recorded in *China Pharmacopoeia* and has been used as food and medicine in Chinese folklore since ancient times [2,3]. Modern pharmacological studies have shown that *P. cyrtonema* rhizome plays an important role in anti-aging, immune function regulation, control of blood glucose and blood lipids, memory improvement, and antitumor and antibacterial effects [4,5,6]. This function is attributed to the abundant functional medicinal components derived from the rhizome, such as polysaccharides and secondary metabolites (e.g., saponins, flavonoids, and alkaloids) [7,8]. Therefore, *P. cyrtonema* rhizome is regarded as an important medicinal and edible food resource and has received increasing attention in recent years in China.

The processing of traditional Chinese medicine (TCM) is a unique traditional pharmaceutical technology in ancient China used to improve the efficacy of TCM, eliminate or reduce the side effects, and change the taste, function, and clinical efficacy of drugs [9]. The purpose of studying processing mechanisms is to explore the scientific basis for processing-induced enhancing or reducing the efficiency, toxicity reduction, and synthesis of new compounds during the whole processing of TCM [10]. For the purpose of detoxification and enhancing medicinal effects, *P. cyrtonema* rhizome (crude medicine) is usually processed using a traditional method known as “nine times of steaming and nine times of drying”, which involves nine cycles of steaming and drying [11,12]. In recent years, several studies have investigated *Polygonatum* rhizome treated with nine processing cycles. These works include examining the structural changes of polysaccharides, analyzing polysaccharide and extract contents, and determining the correlation between processing cycles and the quality characteristics of rhizomes based on color change [12,13,14]. In addition, some findings suggest that nine processing cycles may lead to the loss and change of polysaccharides in *P. cyrtonema* rhizome, which affect factors such as molecular weight, monosaccharide composition, and particle size distribution [15], and processing lasting 18 h lead to the occurrence of various complicated chemical reactions, for example, oxidative decomposition, dehydration, hydrolysis, the Maillard reaction, etc. [16]. Secondary metabolism, which includes a large class of active ingredients with low molecular weight, such as terpenoids, alkaloids, and phenylpropanoids, is derived from the so-called primary metabolism, and has resulted from the interactions between living creatures and environment during the long-term evolution process [17]. These secondary metabolites in plants are important to enhance resistance to stresses and diseases; meanwhile, some of these secondary metabolites have been used in pharmaceutical industries and clinical treatment [18,19,20,21]. However, only a limited number of studies have focused on secondary metabolites in *P. cyrtonema* rhizome to date. Thus, the global analysis on dynamic changes in secondary metabolites in this species during traditional processing remains unclear. In addition, whether this method based on nine cycles of steaming and drying can retain the functional components to their greatest extent is uncertain. Accordingly, a comprehensive investigation into the processing-induced dynamic changes in secondary metabolites and identification of the significantly differentially accumulated secondary metabolites of *P. cyrtonema* rhizome are crucial to evaluate whether nine cycles of processing can effectively enhance the medicinal effects of *P. cyrtonema* rhizome and understand the processing mechanisms for reinforcing medicinal effects.

The field of Plant Metabolomics is based on the Chromatography-Mass Spectrometry (CMS) and Nuclear Magnetic Resonance (NMR) techniques and aims to improve databases for identifying and quantifying all small molecule metabolites in living organisms and biological samples [22]. As an important tool for efficient screening of biologically active substances, metabolomics has been widely used in fields of life science, such as TCM, stress resistance, food nutritional quality, and bio-interactions, and has become an important link between genotypes and phenotypes [23,24,25,26,27,28]. Our current research focuses on the secondary metabolites derived from *P. cyrtonema* rhizome, and metabolomics analysis was performed to investigate the dynamic changes in functional components during traditional processing. Our objectives were to explore the significant changes in functional components induced by traditional processing method and evaluate whether this method based on nine cycles of steaming and drying can effectively reinforce the medicinal effects of *Polygonatum cyrtonema* rhizome. Additionally, our study will provide valuable theoretical and metabolic data to support the pharmacological study of *P. cyrtonema*.

## 2. Results

### 2.1. Global Analysis on Metabolomic Profile of P. cyrtonema Rhizome

**Figure 1:**
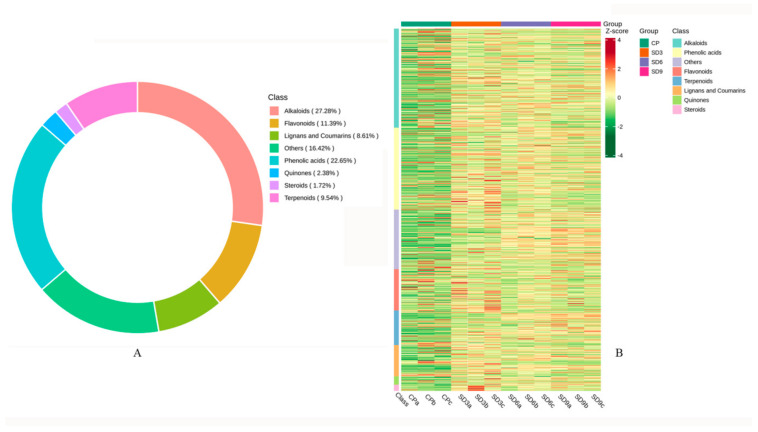
Basic information on secondary metabolites detected in the *P. cyrtonema* rhizomes. (**A**) Quantity and classification of secondary metabolites. (**B**) Clustered heatmap of metabolites in the crude rhizomes (CP group) and in the processed rhizomes (SD3, SD6 and SD9 groups). The number of metabolites was represented with the shades of color. More metabolites were represented with redder shades, while fewer with greener shades.

Based on UPLC-MS/MS and the Metware database, secondary metabolites in the crude *P. cyrtonema* rhizomes (CP group) and the processed *P. cyrtonema* rhizomes (SD3, SD6, and SD9 groups) were obtained. A total of 755 metabolites were tentatively identified from the crude and processed rhizomes, including 206 alkaloids, 171 phenolic acids, 86 flavonoids, 72 terpenoids, 65 lignans and coumarins, 18 quinones, 13 steroids, and 124 others (Figure 1A). This result indicates that the major secondary metabolites in the *P. cyrtonema* rhizomes were phenolic acids and flavonoids, which accounted for 34.04% of the total. Alkaloids, a huge group of low-molecular-weight nitrogen-containing compounds, accounted for 27.28% of the total. The hierarchical clustering heatmap displayed that majority of secondary metabolites in the processed rhizomes (SD3, SD6, and SD9 groups) were more abundant than those in the crude rhizomes (CP group), which indicates that the 3–9 processing cycles of steaming and drying led to a significant global accumulation of secondary metabolites (Figure 1B).

**Figure 2:**
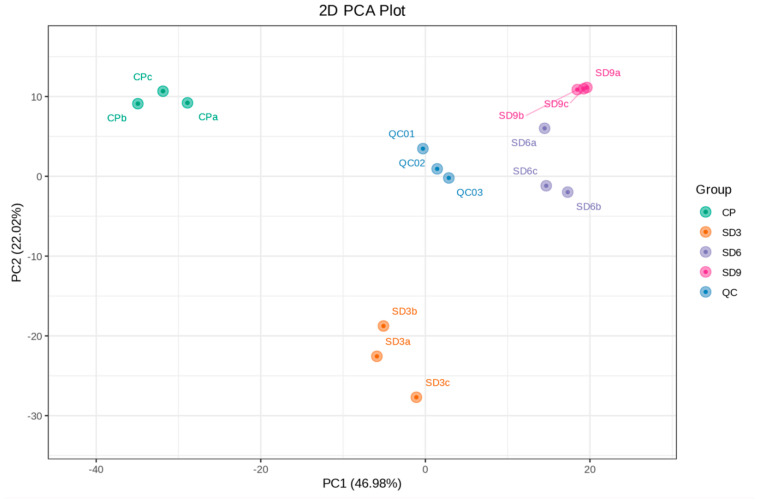
PCA plot of secondary metabolites from 12 *P. cyrtonema* samples. The explained variance ratio of PC1 and PC2 are shown in brackets. CP refers to the crude rhizome samples, and SD3, SD6 and SD9 refer to the processed rhizome samples subjected to three, six, and nine processing cycles, respectively. QC represents the mixture of *P. cyrtonema* rhizome sample extracts for the repeatability test.

The metabolite data of 12 samples were processed based on UV scaling and PCA multivariate statistical analysis to reveal their differences among the four groups and the extent of variability within the same group. In the PCA plot, the explained variance ratio of PC1 was 46.98%, while PC2 was 22.02%. The QC group, derived from three mixed samples and clustering near the center of the PCA plot, showed the same metabolic behavior and thus ensures the stability and repeatability of the entire analysis (Figure 2). In addition, the correlation analyses based on the values of PCC showed that all samples in the same group were close to 1.0 (Figure S1), which indicates that the data from present metabolome analysis were reliable and could be used for following analyses. The PCA plot showed that the three replicates within the SD6 group and three replicates within the SD9 group were not clearly separated and nearly clustered into one group, which implies that the secondary metabolites between the SD6 and SD9 groups had no remarkable differences. The initial findings from the PCA suggest that in the course of the traditional processing of the *P. cyrtonema* rhizomes, the last three cycles of steaming and drying did not induce further significant changes in the abundance of secondary metabolites.

### 2.2. Differentially Accumulated Secondary Metabolite Profiling between Crude and Processed Rhizomes

**Figure 3:**
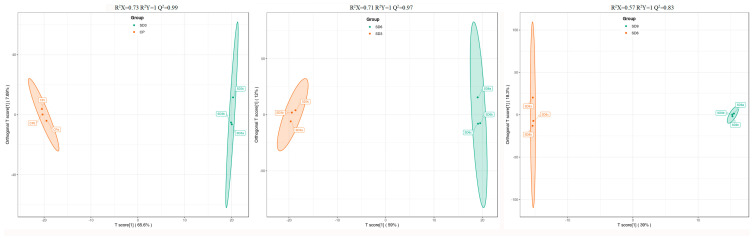
The OPLS-DA score plots of the pairwise comparison group analysis conducted on SD3 vs. CP, SD6 vs. SD3, and SD9 vs. SD6. CP represents the crude rhizome samples and SD3, SD6, and SD9 represent the processed rhizome samples subjected to three, six, and nine processing cycles, respectively.

**Figure 4:**
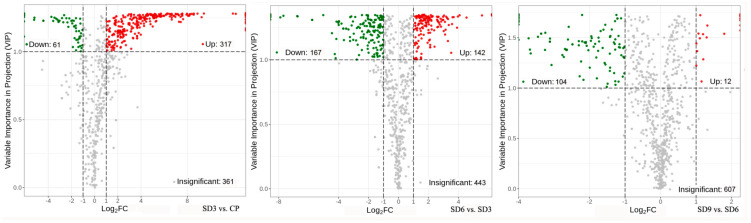
Volcano plots of differentially accumulated secondary metabolites. The dots in plots represent differentially accumulated secondary metabolites, with red dots representing upregulated, green dots representing downregulated, and gray dots indicating insignificant changes.

**Figure 5:**
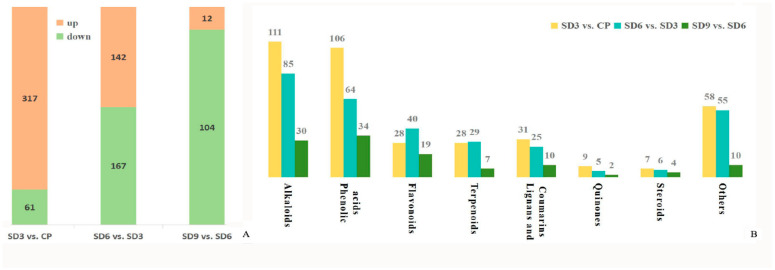
Quantity and class of differentially accumulated secondary metabolites from the three comparison groups. (**A**) Total quantity of differentially accumulated secondary metabolites. (**B**) Specific quantity of differentially accumulated secondary metabolites within each class.

As a multivariate statistical analysis method with supervised pattern recognition, OPLS-DA analysis, was carried out to further reflect the differences among the three groups and identify significantly differentially accumulated secondary metabolites (DASMs) between them. Figure 3 presents OPLS-DA models for the three pairwise comparisons, in which, the values of R^2^Y and Q^2^ for the comparison groups were closer to 1.0, except for the comparison of SD9 vs. SD6 (Q^2^ = 0.83). This result supports that these models were credible and can be used for the further selecting DASMs. The screening standards for DASMs within each pairwise comparison were fold changes ≥ 2.0 [log_2_ (2) ≥1.0] or ≤0.5 [log_2_ (0.5) ≤ −1]) and VIPs ≥ 1.0. The volcano plots in Figure 4 display all DASMs obtained from the three comparison groups. The detailed information on the top 20 DASMs with maximum values of log_2_ (fold change) in each group is listed in Table 1. A total of 378 DASMs (317 up- and 61 down-regulated) were detected from SD3 vs. CP, 309 DASMs (142 up- and 167 down-regulated) from SD6 vs. SD3, and 116 DASMs (12 up- and 104 down-regulated) from SD9 vs. SD6 (Figure 5). Among the top 20 DASMs from each comparison group, all from SD3 vs. CP were upregulated, only 9 from SD6 vs. SD3 were upregulated, and none from SD9 vs. SD6 were upregulated. In addition, among the 378 DASMs in the SD3 vs. CP group, 164 were newly formed metabolites detected only in SD3, while 3 were reduced metabolites detected only in CP; among the 309 DASMs in the SD6 vs. SD3 group, 16 metabolites were detected only in SD6, while 33 only in SD3; among the 116 DASMs in the SD9 vs. SD6 group, 4 metabolites were detected only in SD9, while 30 only in SD6. The detailed information on newly formed and reduced metabolites detected in each comparison group is listed in Table 2 and Table S1.

A large quantity of DASMs were detected from SD3 vs. CP and SD6 vs. SD3, while only a small quantity was detected from SD9 vs. SD6. This finding suggests that significant changes occurred in the content of secondary metabolites from the crude *P. cyrtonema* rhizomes to processed rhizomes subjected to the first six processing cycles, and the last three processing cycles did not induce further significant changes. Moreover, newly formed secondary metabolites were mainly detected in SD3 and reduced secondary metabolites were mainly detected in SD6 and SD9, indicating that the first 3 cycles led to numerous new metabolites while the last 6–9 cycles further reduced the abundance of a small number of original metabolites to undetectable levels. This suggests complex chemical changes occurred in the processed rhizomes. This result about DASMs detection supports that from the PCA analysis and further suggests that the traditional processing method produces a series of chemical changes in the *P. cyrtonema* rhizomes. This phenomenon could make the processed rhizomes significantly different from the crude rhizomes in nutritional and healthy values.

### 2.3. Variation Patterns in the Relative Abundance of Secondary Metabolites

**Figure 6:**
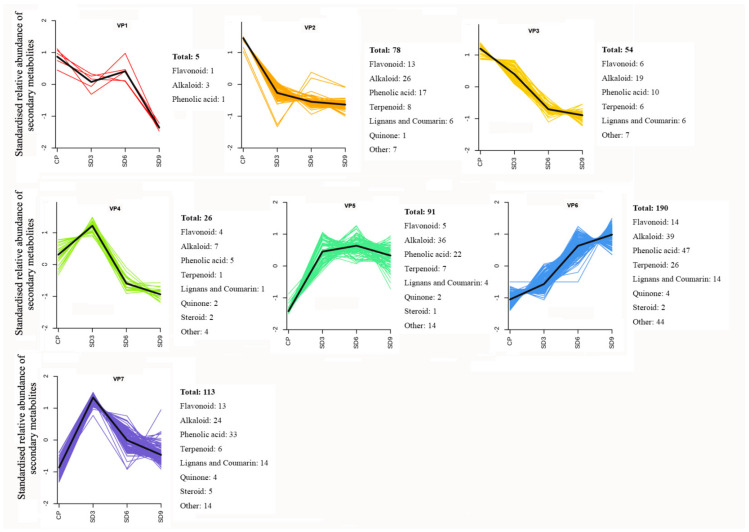
K-Means clustering analysis of secondary metabolites. CP represents the crude rhizome samples and SD3, SD6, and SD9 represent the processed rhizome samples subjected to three, six, and nine processing cycles, respectively.

To learn the variation trends of functional components, K-means clustering analysis was performed for secondary metabolites in all of the *P. cyrtonema* rhizome samples by unit variance scaling on their relative abundance. Based on the standardized variation data during processing, a total of 557 secondary metabolites were grouped into seven variation patterns (Figure 6). The clustering analysis showed that the abundance of most secondary metabolites (420, 75.4%) clustered in variation patterns 4–7 and increased in SD3 samples compared to CP samples. Only 137 DASMs (24.6%) clustering in variation patterns 1–3 decreased in SD3 samples compared to CP samples. Therefore, for most secondary metabolites in the crude *P. cyrtonema* rhizomes, the first three processing cycles of steaming and drying significantly enhanced their accumulation in abundance. However, the first three processing cycles also induced a certain level of chemical changes in a small number of secondary metabolites, which led to a significant decrease in their abundance.

In all of the 557 secondary metabolites with seven different variation patterns, most exhibited four change trends in abundance during processing. Among them, 34.11% presented as continuously increasing from CP to SD9, which mainly consisted of alkaloids, phenolic acids, and terpenoids (VP6); 23.69% showed a continuous decrease from CP to SD9, which primarily comprised alkaloids, phenolic acids, and flavonoids (VP2 and VP3); 24.96% demonstrated a trend of first increasing from CP to SD3 and then decreasing from SD3 to SD9, which mainly included alkaloids, phenolic acids, and flavonoids (VP4 and VP7); and 16.34% exhibited a trend of first significantly increasing from CP to SD3, then slightly rising from SD3 to SD6, and last slightly decreasing from SD6 to SD9, which primarily consisted of alkaloids and phenolic acids (VP5). Therefore, alkaloids, phenolic acids, and flavonoids were the main secondary metabolites with diversified variation patterns during the nine processing cycles. In addition, nearly half of the terpenoid compounds, which were mainly diterpenoids, presented a continuously increasing trend from CP to SD9, which indicates that continued processing from 1–9 cycles led to continuous accumulation in their abundance.

### 2.4. Annotation and Functional Classification of Secondary Metabolites

**Figure 7:**
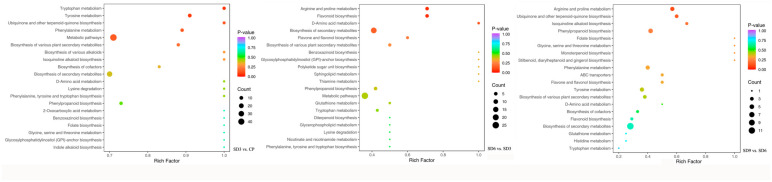
The most significantly enriched KEGG pathways ranked from 1–20 according to their *p*-value. The higher or lower *p*-value was represented with the color of the dots. The size of a dot refers to the number of annotated differentially accumulated secondary metabolites. Higher *p*-values were represented with bluer dots, while lower *p*-values were represented with redder dots. More metabolites were represented with bigger dots, while fewer metabolites were represented with smaller dots.

Enrichment analysis was carried out on all of the identified secondary metabolites to further identify the KEGG pathways in which the differentially accumulated secondary metabolites were significantly enriched. A total of 289 secondary metabolites were mapped to the KEGG database categories and annotated in metabolic pathways, with 94 from SD3 vs. CP, 99 from SD6 vs. SD3, and 96 from SD9 vs. SD6 being annotated to 38, 29, and 22 significantly enriched pathways, respectively. Among all of the 289 annotated secondary metabolites, 125 were DASMs, including 63 from SD3 vs. CP, 34 from SD6 vs. SD3, and 28 from SD9 vs. SD6 (Table S2). The most significantly enriched KEGG pathways (ranked from 1–20 according to their *p*-value) in the three comparison groups are presented in Figure 7, most of which were related to the biosynthesis of various secondary metabolites, including alkaloids, flavonoids, and terpenoids, as well as the metabolisms of many kinds of amino acids, such as lysine degradation and biosynthesis of amino acids (Table 3). Meanwhile, most DASMs involved in these pathways were upregulated after the first three processing cycles. However, after six processing cycles, nearly half of the DASMs were related to the synthesis of various secondary metabolites and most of these DASMs were involved in the metabolisms of amino acids were downregulated. After the nine processing cycles, nearly all of the DASMs enriched in these pathways were downregulated. The KEGG enrichment analysis supports the results obtained from DASMs and variation pattern analysis, which indicates that the first three processing cycles significantly enhanced the synthesis of various secondary metabolites. However, these enhancing effects were gradually reversed by the continuation to 6–9 processing cycles. In addition, the processing significantly affected the metabolisms of many kinds of amino acids, which suggests significant changes in the chemical synthesis or degradation of amino acids.

### 2.5. Processing-Induced Changes in the Functional Components of P. cyrtonema Rhizome

**Figure 8:**
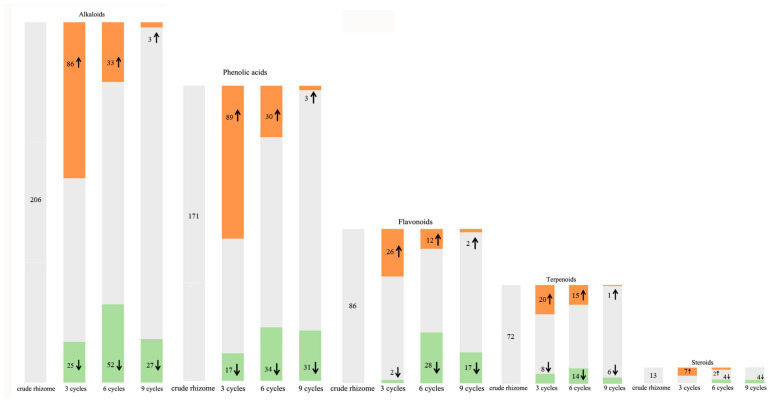
Changes in relative abundance of functional components in the *P. cyrtonema* rhizomes during nine processing cycles. The shades of color and arrows represent up- or down-regulated components, with orange shades and ↑ representing upregulated, green shades and ↓ representing downregulated, and gray shades representing unchanged. The number represent the quantity of up- or down-regulated and unchanged components.

**Figure 9:**
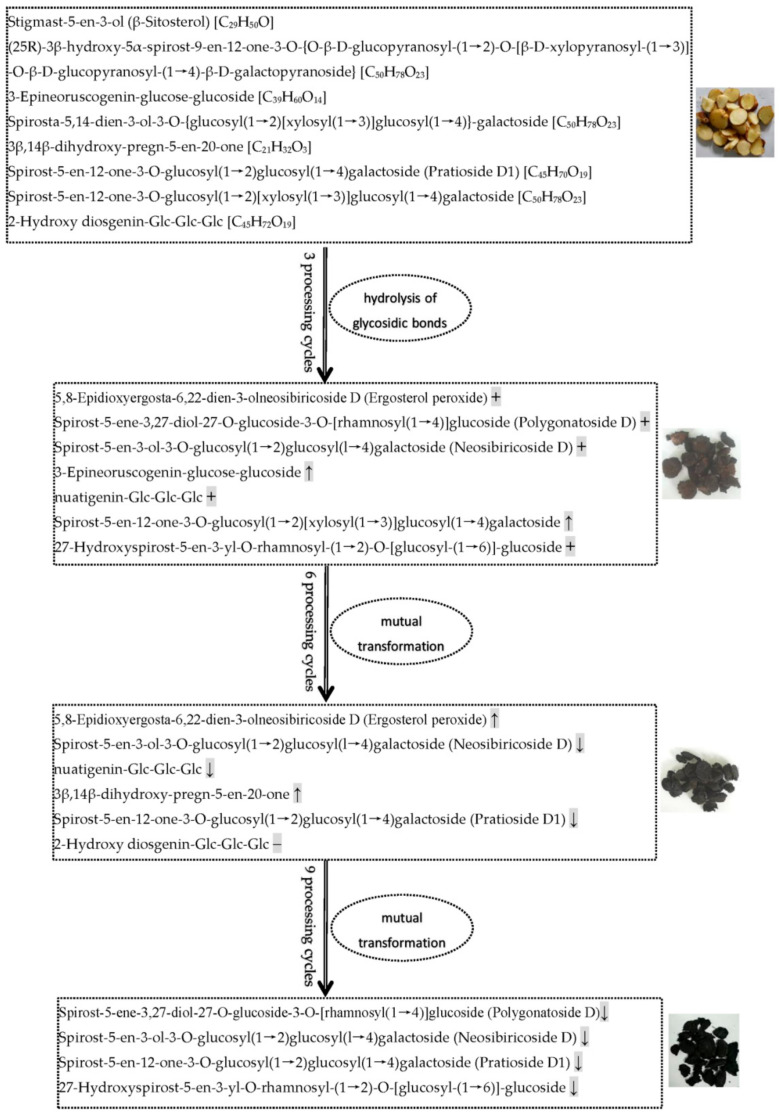
Processing-induced chemical changes on steroids in the *P. cyrtonema* rhizomes during the nine cycles of steaming and drying. The arrow ↑ and ↓ refer to the steroids with increased and decreased relative abundance, respectively. The symbol + and − represent the newly formed and reduced steroids, respectively.

Alkaloids, phenolic acids, flavonoids, terpenoids, and steroids were selected to determine processing-induced changes in the key functional components of *P. cyrtonema* rhizome, and changes in their abundance during processing were further analyzed. The results showed that continued processing of steaming and drying induced global changes (up- and down-regulated) in the abundance of these functional components. As for alkaloids and phenolic acids (Figure 8), some of them were up- or down-regulated in abundance after the first three processing cycles. However, with increased processing cycles from 6–9, the number of upregulated metabolites decreased gradually, and downregulated metabolites increased gradually. With regard to flavonoids, terpenoids, and steroids (Figure 8), some of them were upregulated in abundance after the first three processing cycles. Similarly, with increased processing cycles from 6–9, the number of upregulated metabolites decreased gradually, and downregulated metabolites began to occur. Among these regulated functional components, the most abundant were alkaloid, phenolamine, and plumerane, belonging to the alkaloid class; flavones, flavonols, and flavonoids, belonging to the flavonoid class; monoterpenoids belonging to the terpenoid class; and steroidal saponins belonging to the steroid class. A total of eight steroids were identified from the crude rhizomes, and the significant chemical changes, such as hydrolysis of glycosidic bonds and mutual transformation, on these steroids during the nine cycles of steaming and drying are presented in Figure 9. These results suggest that processing for a short period of time can significantly induce enhancing effects, which will promote the chemical synthesis of functional components in *P. cyrtonema* rhizome. However, these enhancing effects were unavoidably reversed or weakened under continued processing for a long period of time. Accordingly, the traditional processing period of steaming and drying should be reduced from 9 cycles to 3–6 cycles to maintain enhancing effects on the accumulation of functional components. In addition, different functional components, even if belonging to the same class, have different response mechanisms toward processing, which are presented as upregulated, downregulated, or stable in their abundance.

## 3. Discussion

Complex chemical changes have occurred gradually during the process of TCM processing. Newly formed chemical constituents may be the basis of clinical efficacy. The main chemical reactions presented in processing are hydrolysis reactions, oxidation reactions, replacement reactions, isomerization reactions, decomposition reactions, etc. [29]. With some TCM, such as the root of *Aconitum* and the rhizomes of *Panax ginseng* and *Rhei radix*, it has been proven that processing induces their distinct chemical variations, meanwhile reducing the toxicity and changing the therapeutic effect [10]. Understanding these complicated processing mechanisms provides a theoretical and metabolic data basis for improving processing techniques and for the pharmacological study of *P. cyrtonema*.

In the processed *P. cyrtonema* rhizomes, we observed significant abundance changes of secondary metabolites and detected numerous newly formed secondary metabolites and a small number of reduced secondary metabolites. These findings suggest traditional processing method results in complex changes in functional components of *P. cyrtonema* rhizome, and multiple structural transformation mechanisms of secondary metabolites for enhancing the medicinal effects might be involved in the processing. For example, the four detected steroidal saponins, including polygonatoside D, neosibiricoside D, nuatigenin-Glc-Glc-Glc and 27-hydroxyspirost-5-en-3-yl-*O*-rhamnosyl-(1→2)-*O*-[glucosyl-(1→6)]-glucoside, exhibited a trend of first increasing drastically and then decreasing in their abundance during processing (VP7 in Figure 6), indicating that the first three cycles increased the content of sapogenins by catalyzing the hydrolysis of glycosidic linkages. Then, mutual transformation resulted in a decrease during the last 6–9 cycles. With regard to flavonoids, polygonatone C continuously decreased in abundance (VP3 in Figure 6), while vicenin-2, apigenin-7-*O*-(6″-*p*-coumaryl)-glucoside, rhoifolin, and isovitexin exhibited a trend of first increasing slowly and then decreasing during processing (VP4 in Figure 6), which could have resulted from hydrolysis reactions and transformation taking place in flavonoid glycosides and flavonoid aglycones, respectively. In addition, as a saccharide derivative, 5-hydroxymethylfurfural (5-HMF) is produced by dehydration of hexose under acidic and thermal conditions. The detected 5-HMF in the processed rhizomes continuously increased in abundance (VP6 in Figure 6), suggesting that a Maillard reaction derived from sugar–amino acid in the processed rhizomes happened to form furfural derivatives. Hence, our present study suggests that processing induced multiple structural transformations in the *P. cyrtonema* rhizomes, which also were in agreement with other studies. Liang et al. (2022) observed that with an increase in processing time drastic changes occurred in the chemical components of *P. cyrtonema* rhizome, such as component isomerization, decreased type and content of primary glycosides, and increased content of aglycones [30]. Jiang et al. (2022) found that during a 18-h processing of steaming various complicated chemical reactions occurred, for example, oxidative decomposition, dehydration, hydrolysis, the Maillard reaction, etc., which consequently resulted in significant qualitative and quantitative changes in chemical ingredients such as fatty acids, saponins, flavonoid, oligosaccharides, and polysaccharides [16]. Therefore, increasing the content of functional components is an important processing mechanism related to the enhancing the medicinal effects of *P. cyrtonema* rhizome. In addition, the structural transformation of functional components from crude to processed rhizomes might also be an equally important mechanism for enhancing the medicinal effects. Furthermore, the structural transformation-mediated synthesis of new functional components mainly occurred during the first 3 processing cycles, while reduction of original components mainly occurred during the last 6–9 processing cycles.

Apart from the structural transformation of components, directly reducing the contents of toxic components is also a main processing goal in Chinese herbal medicines [10]. Alkaloids are the largest class of secondary metabolites in nitrogenous organic compounds. Some alkaloids are mainly present in several plant families related to TCM including Ranunculaceae, Menispermaceae, Papaveraceae, Solanaceae, Loganaceae, Berberidaceae, Leguminosae, etc. [31]. Extracts from alkaloid-containing plants have been used the clinical treatment for bite injury, influenza with fever, and mental confusion. However, in spite of their remarkable health benefits and promising application value in pharmaceutical industries, the toxicity of some types of plant alkaloids, for example, pyrrolizidine, tropane, piperidine, indolizidine, and steroidal alkaloids, has been observed in animals and humans [32,33]. Alkaloid compounds were found to be the most abundant secondary metabolites in the *P. cyrtonema* rhizomes. Among the 206 alkaloids compounds, 20 of them were regarded as potential toxic alkaloids according to the above-mentioned compound types reported in the literature, including seven piperidine alkaloids, seven pyridine alkaloids, two isoquinoline alkaloids, two tropan alkaloids, and two steroidal alkaloids (Table S3). Continued processing did not lead to the complete decomposition of these alkaloids, and only the abundance of piperidine and 6-deoxyfagomine (piperidine alkaloids) in the rhizomes processed for six cycles were significantly decreased compared to the crude rhizomes (VP3 in Figure 6). Although, the toxicity of the crude *P. cyrtonema* rhizomes and related toxic components in them are unclear to date, our present study suggested that reducing the contents of potential toxic alkaloids and decomposing toxic alkaloids may not be the main mechanisms for the detoxification of *P. cyrtonema* rhizome. Therefore, we presume some chemical changes or physically structural alterations may have occurred during processing which were related to the detoxification of the *P. cyrtonema* rhizome.

Flavonoids, widely present in various plants, are a diverse class of polyphenolic compounds with antioxidant, hypoglycemic, hypolipidemic, and anticancer properties [34]. In the rhizomes of *Polygonatum* plants, flavonoids and their glycosides are regarded as important functional medicinal components. Currently, 61 flavonoids have been isolated from *Polygonatum* plants, with 13 of them identified in *P. cyrtonema* according to Zhang et al. (2019) [5]. The stability of different flavonoids, such as flavanones, flavonols, and flavones, is influenced not only by the processing but also by the flavonoid structure [35]. Jiang et al. (2022) identified 17 flavonoids including 15 homoisoflavonoids and 2 flavonols [16]. Liang et al. (2022) identified 12 flavonoids from the processed *P. cyrtonema* rhizomes, and found vitexin 2″-*O*-xyloside, a flavonoid, completely disappeared after seven processing cycles [30]. In our study, a total of 86 flavonoids were detected, which were mainly flavones, flavonols, isoflavones, chalcones, and other types of flavonoids including 16 homoisoflavonoids (Table S4). Homoisoflavones are characteristic components with a wide range of biological activities and a quality marker of *Polygonatum* plants [36]. Therefore, our finding enriches the data on homoisoflavonoids in *Polygonatum* plants. Continued processing induced diversified variation patterns in the abundance of different flavonoids, as well as the synthesis of new flavonoids and the reduction of original flavonoids. The changed functional components observed in most flavonoids suggest that 3–6 processing cycles can enhance medicinal effects by increasing their contents and inducing the synthesis of 85% of the new flavonoids. Continued processing until 9 cycles will unavoidably weaken the enhancing effect by decreasing the increased contents during 3–6 processing cycles and further reducing more flavonoids.

Phenolic acids, which are a subclass of plant phenolics, exhibit tremendous antioxidant activity and protective effects, including antimicrobial, anticancer, anti-inflammatory, and anti-mutagenic effects [37]. In this study, a total of 171 phenolic acids were detected, among which 106 were DASMs. As the main secondary metabolites rich in the *P. cyrtonema* rhizomes, the variation patterns exhibited by most of them suggest that three processing cycles induced their significant accumulation and the addition of 91% of the new phenolic acid compounds. The similar behavior was reported by a study by Yao et al. (2022) with an increasing trend in the abundance of 15 identified phenolic acids from *P. cyrtonema* rhizomes subjected to three processing cycles and a slowing down of the change rate under continued processing [15].

Saponins in medicinal plants exhibit essential medicinal properties, such as anti-inflammatory, antiviral, insecticidal, and anticancer actions [38]. Saponins are primarily classified as two kinds, steroid and triterpenoid saponins, according to their different structure of the hydrophobic aglycone unit [39]. As the primary active ingredient, *Polygonatum* plants are rich in steroidal saponins, and 10 steroidal saponins have been isolated and identified from *P. cyrtonema* according to the report by Shi et al. [40]. In addition, Jiang et al. (2022) identified 38 saponins and Liang et al. (2022) identified 19 saponins from the processed *P. cyrtonema* rhizomes [16,30]. In our study, a total of 13 steroidal saponins and 3 triterpene saponins were detected, among which spirost-5-en-12-one-3-*O*-glucosyl(1→2)glucosyl(1→4)galactoside (Pratioside D1) (Formula: C_45_H_70_O_19_) was also reported by Liang et al. (2022) and Jiang et al. (2022) [16,30]. The variation patterns suggest that first three processing cycles were sufficient to increase the contents of saponins and induce 100% of the new saponin compounds. Conversely, continued processing for more than three cycles will unavoidably weaken the enhancing effects induced by the first three cycles. Particularly, a saponin compound, 2-Hydroxy diosgenin-Glc-Glc-Glc (Formula: C_45_H_72_O_19_) was reduced to undetectable level after six processing cycles.

Seventeen kinds of amino acids have been detected in crude *Polygonatum* rhizomes, which were important flavor and nutritional components [41]. However, the effects of traditional processing by steaming and drying on the composition of amino acids, contents, and nutritional benefits of *Polygonatum* plants as edible food have not been fully understood to date. KEGG enrichment analysis suggests that processing has significant effects on amino acid metabolisms. Therefore, in future work, changes in amino acid metabolisms during processing will be explored to further provide metabolic data support for the nutritional and healthy values of the processed *P. cyrtonema* rhizomes from the prospective of amino acid nutrients.

Our present study based on metabolomics reveals complicated changes in secondary metabolites of *P. cyrtonema* rhizome during the traditional processing of “nine-steaming and nine-drying”. Undoubtedly, processing produces beneficial effects on reinforcing medicinal effects by promoting the accumulation of functional components and inducing the formation of novel components and on reducing toxicity to a certain extent by decreasing the contents of toxic components or inducing the structural transformation of potential toxic components, which is meaningful for edible safety and increasing the medicinal values of *P. cyrtonema* rhizome. However, processing also brings adverse effects, given that it can significantly decrease the contents of a small number of functional components in the rhizome, even after only three cycles. Apart from these functional components belonging to secondary metabolites, polysaccharides are also a main functional component in *P. cyrtonema* rhizome. Previous studies have found the highest content of polysaccharides in crude rhizomes, with decreasing content in processed rhizomes with increasing processing cycles, and four processing cycles may be a seasonable method for processing *Polygonatum* rhizomes to maintain more bioactive ingredients [15,42]. Based on the comprehensive quality evaluation index, five processing cycles were regarded as a turning point with the highest index [12]. Therefore, achieving the purpose of reinforcing medicinal effects, reducing toxicity by processing, and preserving functional components as much as possible in processed *P. cyrtonema* rhizomes should be comprehensively considered. Thus, we propose that the traditional processing method should be improved by changing the processing period. Furthermore, the 9 processing cycles of steaming and drying used in the processing of *P. cyrtonema* rhizomes might need to be reduced to 3–6 cycles.

Processed *P. cyrtonema* rhizomes are widely manufactured as health foods such as health candy and health beverages including health tea and health wine and are also used as TCM for clinical therapy. Although processed TCMs are widely used, the key scientific mechanism of processing is not clarified for most TCMs, including *Polygonatum* plants, to date. During the process of heating and drying, complicated changes in functional components of *P. cyrtonema* rhizome may occur not only in regulated contents but also in formed novel components. In most circumstances, the changes on contents and composition may be performed simultaneously. Future studies should be devoted to comprehensively elucidating the processing-induced scientific mechanisms for reinforcing medicinal effects and reducing toxicity. Thus, additional efforts should be made to investigate the association of chemical and pharmacological changes using advanced technologies and assess the contribution of processing-induced chemical alteration to the changed bioactivities of *P. cyrtonema*. Furthermore, a unified and scientific processing technology should be established in the future to improve the traditional processing method used in the processing of *P. cyrtonema*.

## 4. Materials and Methods

### 4.1. Plant Material Selection and Pre-Treatment

Five-year-old *Polygonatum cyrtonema* Hua. plants were collected from plantations in Quzhou, Zhejiang Province (Southeast China). The rhizomes were removed from the plants and cleaned with water. Then, the rhizomes of 3 *P. cyrtonema* plants were mixed to form a biological replicate. The whole rhizomes were divided uniformly into two groups: one as crude rhizome (CP group, non-processed) with three replicates, and the other stored at 4 °C for a short period prior to follow-up with the “nine-steaming and nine-drying” method to form the processed rhizomes (SD groups) with three replicates. The samples in the CP group were further cut into slices and put in liquid nitrogen for quick freezing, and stored in a ultra-low temperature freezer (−80 °C) until further analysis.

### 4.2. Polygonatum Rhizome Processing

**Figure 10:**
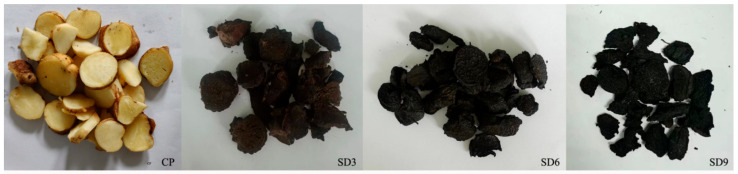
The *P. cyrtonema* rhizome samples during nine processing cycles of steaming and drying. CP represents the crude rhizome samples and SD3, SD6, and SD9 represent the processed samples subjected to three, six, and nine processing cycles, respectively.

The processing of *P. cyrtonema* rhizomes followed a method described previously by Guo et al. (2022) [14]. In brief, the fresh *P. cyrtonema* rhizomes were placed in a steamer to steam for 6 h, subsequently moisturized overnight, and then dried in an oven at 50 °C for 8 h. This procedure was repeated for 1–9 cycles. Next, rhizomes subjected to 3, 6, and 9 cycles of processing were collected as the SD3, SD6, and SD9 groups, respectively. Similarly, the samples in the SD groups were further cut into slices, and put in liquid nitrogen for quick freezing, and stored in a ultra-low temperature freezer (−80 °C) until further analysis (Figure 10).

### 4.3. Sample Preparation for Metabolome Analysis

Sample preparation and extraction followed the methods provided by Metware Biotechnology Co., Ltd. (Wuhan, China). Briefly, the *P. cyrtonema* rhizome samples were freeze-dried in a freeze dryer (Scientz-100F) using vacuum freeze-drying technology, followed by grinding for 1.5 min at 30 Hz with a grinder (MM 400, Retsch, Haan, Germany). Then, 50 mg of powder from each sample was weighted and dissolved in 1.2 mL of 70% methanolic aqueous solution pre-cooled at −20 °C by vortex shaking for a short time, and then stored overnight at 4 °C. After extraction, the mixtures were centrifuged at 13,400× *g* for 3 min, and the supernatant was collected and filtered through a microporous membrane (SCAA-104, 0.22 μm pore size; ANPEL, Shanghai, China). Finally, the filtered samples were stored in injection vials for metabonomic analysis. The extractions of the CP, SD3, SD6, and SD9 samples were performed in triplicate. For testing the repeatability of extraction and detection, three QC samples were prepared by mixing all of the *P. cyrtonema* rhizome sample extracts together.

### 4.4. UPLC-MS/MS and ESI-Q TRAP-MS/MS

The extracts of the *P. cyrtonema* rhizome samples were detected by applying the ultra-performance liquid chromatography electrospray ionization tandem mass spectrometry system [UPLC-ESI-MS/MS, UPLC, ExionLC™ AD, https://sciex.com.cn (accessed on 10 september 2023)] and electrospray ionization-triple quadrupole-linear ion trap mass spectrometry system (ESI-Q TRAP-MS/MS). The UPLC analytical conditions and the ESI source operation parameters were described according to a previously published method used by our laboratory [43].

### 4.5. Metabolite Annotation

The mass spectrometry data obtained by using targeted MRMs were subjected to qualitative and quantitative analysis according to a previously published method used by our laboratory based on the Metware database (MWDB v2.0, Wuhan, China) [43]. During the qualitative analysis, non-target repeat signals were eliminated according to the information of spectra of metabolites matching the MWDB of secondary metabolites, just as described by Chen et al. (2013) and Zhu et al. (2018) [44,45]. The chromatographic peak area, integral data of the metabolites, were derived and used to calculate their relative abundance [46,47].

### 4.6. Multivariate Statistical Analysis

Multivariate statistical analyses were performed according to a previously published method used by our laboratory [43]. Briefly, metabolite data were log_2_-transformed and underwent autoscaling before any statistical analysis. Metabolite data from all of the rhizome samples were used for PCA, HCA, and OPLA-DA analyses by using the Metware Cloud [https://cloud.metware.cn (accessed on 8 November 2023)]. OPLS-DA analysis referred to a previously published method by Thévenot et al. (2015) [48].

The DASMs were detected by variable importance in project scores (VIP ≥ 1) and fold change ≥ 2 or fold change ≤ 0.5, with VIP values extracted from the OPLS-DA results. The DASMs were annotated in pathways by using the KEGG database [http://www.kegg.jp/kegg/compound (accessed on 12 November 2023)]. Pathways with significantly regulated metabolites were further subjected to metabolite set enrichment analysis, and their significance was determined by hypergeometric test’s *p*-values.

## 5. Conclusions

From the results obtained, it can be concluded that processing significantly increased the global accumulation of secondary metabolites in the processed *P. cyrtonema* rhizomes compared to the unprocessed crude rhizomes. Concretely, traditional processing lasting three cycles of steaming and drying greatly enhanced the contents of functional components in the rhizomes and induced the formation of numerous new components. However, the enhancing effects were unavoidably reversed or weakened under continued processing lasting 6–9 cycles. Meanwhile, continued processing also reduced the contents of a small number of original components to undetectable level. Therefore, processing led to complicated changes in functional components in the *P. cyrtonema* rhizomes, and processing can effectively reinforce medicinal effects by two main mechanisms, enhancing chemical synthesis and inducing structural transformation of functional components in a short period of time. Considering that no significant changes have occurred in the functional components after 6 processing cycles of steaming and drying, the traditional processing method might need to be reduced to 3–6 cycles for achieving optimal effects on enhancing chemical synthesis and inducing structural transformation of functional components, thus reinforcing the medicinal effects of *P. cyrtonema* rhizome.

## Figures and Tables

**Table 1:** The detailed information of the top 20 differentially accumulated secondary metabolites with maximum values of Log_2_FC.

Class	DASM//Log_2_ FC		
	SD3 vs. CP	SD6 vs. SD3	SD9 vs. SD6
Alkaloids	Cephalanthrin A//12.57 1-Acetyl-β-carboline//8.98 (R)-1,2,3,4-Tetrahydro-3-carboxy-2-carboline//8.52 N-benzoyl-2-aminoethyl-β-D-glucopyranoside//8.18 Valerine//7.27 (1R,3S)-1-Methyl-1,2,3,4-tetrahydro-β-carboline-3-carboxylic acid//6.6	4′-*O*-Methylnorbelladine//4.14 Casuarine analogue//−3.2 Folicanthine//−3.66	
Phenolic acids	Chlorogenic acid methyl ester//9.32 4-*O*-Caffeoylquinic acid methyl ester//7.92 3-hydroxyphenylacetic acid//7.38 methyl 5-caffeoylquinate//6.91 4-Hydroxybenzoic acid//6.87 1-*O*-Feruloylquinic acid//6.8 2,3-Dihydroxybenzoic acid//6.54	Ethyl malto//4.14 Antiarol; 3,4,5-Trimethoxyphenol//3.64 methyl 5-caffeoylquinate//−3.33 1-*O*-Feruloylquinic acid//−3.41 3-*O*-Feruloylquinic acid//−3.48 4-*O*-Caffeoylquinic acid methyl ester//−3.64 *p*-Hydroxyphenyl 6-*O*-(E)-caffeoyl-β-D-allopyranoside//−4.7	1-*O*-*p*-Coumaroylquinic acid//−2.64 4-*O*-Caffeoylquinic acid methyl ester//−2.8 Chlorogenic acid methyl ester//−2.85 1-*O*-Feruloylquinic acid//−2.9 2-Hydroxycinnamic acid//−3.04 2-(Formylamino)benzoic acid//−3.13 α-Hydroxycinnamic Acid//−3.28 3-Hydroxycinnamic Acid//−3.33 Methyl 5-caffeoylquinate//−3.43 3,4,5-Trimethoxycinnamic acid//−3.73
Flavonoids	Sesuvioside A//7.41	3-Hydroxy-4′,5,7-Trimethoxyflavanone//4.03 Butin; 3′,4′-Trihydroxyflavanone//3.41 Sesuvioside A//−3.25 Isorhamnetin-3-*O*-neohesperidoside//−3.73 3,5,7-Trihydroxy-6,8-dimethyl-3-(4′-hydroxybenzyl)-chroman-4-one (Polygonatone C)//−4.23	3-[(3,4-dihydroxyphenyl)methylidene]-5,7-dihydroxy-6-methoxy-2h-1-benzopyran-4-one glucosyl rhamnoside//−2.72 Apigenin-6-*C*-(2″-glucosyl)arabinoside//−2.78 Tricin (5,7,4′-Trihydroxy-3′,5′-dimethoxyflavone)//−2.95
Steroids		Spirost-5-en-12-one-3-*O*-glucosyl(1→2)glucosyl(1→4)galactoside (Pratioside D1)//−4.0	Spirost-5-ene-3,27-diol-27-*O*-glucoside-3-*O*-[rhamnosyl(1→4)]glucoside (Polygonatoside D)//−2.86 27-Hydroxyspirost-5-en-3-yl-*O*-rhamnosyl-(1→2)-*O*-[glucosyl-(1→6)]-glucoside//−3.17 Spirost-5-en-3-ol-3-*O*-glucosyl(1→2)glucosyl(l→4)galactoside (Neosibiricoside D)//−3.32
Lignans and coumarins	Phellodenol E//8.25 Guaiacylglycerol-β-guaiacyl ether//6.69	7,8-Dihydroxy-4-methylcoumarin//5.33 7-Hydroxycoumarin; Umbelliferone//3.38 5,7-dihydroxy-4-phenylcoumarin//3.22	Phellodenol E//−3.12
Others	2,5-Dihydroxybenzaldehyde//7.4 Protocatechualdehyde//6.96 4-Methyl-5-thiazoleethanol//6.75 4-Hydroxybenzaldehyde//6.46	Squamocin K//3.56	4-hydroxyphenyl acrylaldehyde//−3.67 4-Methylbenzaldehyde//−3.92 3-Methylbenzaldehyde//−4.11
Total	Up: 20; down: 0	Up: 9; down: 11	Up: 0; down: 20

Abbreviation: DASM, differentially accumulated secondary metabolite.

**Table 2:** The number of newly formed and reduced secondary metabolites detected from the three pairwise comparison groups.

Secondary Metabolites	Comparison Group	Total Number	Class							
			Alkaloids	Phenolic Acids	Flavonoids	Terpenoids	Lignans and Coumarins	Quinones	Steroids	Others
Newly formed	SD3 vs. CP	164	45	42	23	13	15	8	5	13
	SD6 vs. SD3	16	6	3	4		1			2
	SD9 vs. SD6	4		1		1				2
Reduced	SD3 vs. CP	3	1		1		1			
	SD6 vs. SD3	33	10	6	11	1	1		1	3
	SD9 vs. SD6	30	9	10	4	2	3	1		1

Note: In the SD3 vs. CP group, newly formed secondary metabolite refers to its relative abundance in CP samples being undetectable, while reduced secondary metabolite refers to its relative abundance in SD3 samples being undetectable; in the SD6 vs. SD3 group, newly formed secondary metabolite refers to its relative abundance in SD3 samples being undetectable, while reduced secondary metabolite refers to its relative abundance in SD6 samples being undetectable; in the SD9 vs. SD6 group, newly formed secondary metabolite refers to its relative abundance in SD6 samples being undetectable, while reduced secondary metabolite refers to its relative abundance in SD9 samples being undetectable.

**Table 3:** Significantly enriched KEGG pathways related to the synthesis of secondary metabolites and amino acid metabolisms.

Comparison Group	Biosynthesis of Secondary Metabolites	Number of DASMs	Amino acid Metabolism	Number of DASMs
SD3 vs. CP	Ubiquinone and other terpenoid-quinone (ko00130)	4↑; 1↓	Tryptophan (ko00380)	4↑; 3↓
	Various plant secondary metabolites (ko00999)	5↑; 2↓	Tyrosine (ko00350)	7↑; 3↓
	Various alkaloids (ko00996)	3↑	Phenylalanine (ko00360)	8↑
	Isoquinoline alkaloid (ko00950)	3↑	D-Amino acid (ko00470)	2↓
	Secondary metabolites (ko01110)	23↑; 5↓	Lysine degradation (ko00310)	1↑; 1↓
	Indole alkaloid (ko00901)	1↑	Phenylalanine, tyrosine and tryptophan (ko00400)	1↑; 1↓
	Monoterpenoid (ko00902)	1↑	Glycine, serine and threonine (ko00260)	1↑
	Diterpenoid (ko00904)	1↓	Lysine (ko00300)	1↑
	Isoflavonoid (ko00943)	1↑	Biosynthesis of amino acids (ko01230)	1↑
	Flavonoid (ko00941)	2↑	Histidine (ko00340)	2↑
	Tropane, piperidine and pyridine alkaloid (ko00960)	1↑; 2↓	Arginine and proline (ko00330)	2↓
SD6 vs. SD3	Flavonoid biosynthesis (ko00941)	5↑	D-Amino acid (ko00470)	2↓
	Secondary metabolites (ko01110)	9↑; 9↓	Tryptophan (ko00380)	3↓
	Flavone and flavonol (ko00944)	3↓	Phenylalanine, tyrosine and tryptophan (ko00400)	1↓
	Various plant secondary metabolites (ko00999)	1↑; 3↓	Histidine (ko00340)	1↑
	Diterpenoid (ko00904)	1↑	Tyrosine (ko00350)	1↑; 1↓
	Tropane, piperidine and pyridine alkaloid (ko00960)	2↓	Phenylalanine (ko00360)	1↑
	Isoflavonoid (ko00943)	1↑		
	Isoquinoline alkaloid (ko00950)	1↓		
	Ubiquinone and other terpenoid-quinone (ko00130)	1↓		
SD9 vs. SD6	Ubiquinone and other terpenoid-quinone (ko00130)	3↓	Arginine and proline (ko00330)	4↓
	Isoquinoline alkaloid (ko00950)	2↓	Glycine, serine and threonine (ko00260)	1↓
	Monoterpenoid (ko00902)	1↓	Phenylalanine (ko00360)	4↓
	Flavone and flavonol (ko00944)	2↓	Tyrosine (ko00350)	4↓
	Various plant secondary metabolites (ko00999)	1↑; 2↓	D-Amino acid (ko00470)	1↓
	Flavonoid (ko00941)	1↑; 1↓	Histidine (ko00340)	1↓
	Secondary metabolites (ko01110)	2↑; 10↓	Tryptophan (ko00380)	1↓
	Tropane, piperidine and pyridine alkaloid (ko00960)	1↓		

↑: upregulated DASMs; ↓: downregulated DASMs.

## Data Availability

The original contributions presented in the study are included in the article/Supplementary materials, further inquiries can be directed to the corresponding authors.

## Supplementary Materials

The following supporting information can be downloaded at: https://www.mdpi.com/article/10.3390/ph17081074/s1, Figure S1: The correlation assessment of biological replicates from the *P. cyrtonema* rhizome samples. The value of Pearson’s correlation coefficient (*PCC*) was represented with the shades of color. Higher values were represented with redder shades, while lower values were represented with greener shades; Table S1: Newly formed and reduced secondary metabolites in the three comparison groups; Table S2: KEGG pathways and annotated compounds in the three comparison groups; Table S3: Potential toxic alkaloids identified in this study; Table S4: Homoisoflavonoids identified in this study.
