# Supplementary material for: Traditional Processing Can Enhance the Medicinal Effects of Polygonatum cyrtonema by Inducing Significant Chemical Changes in the Functional Components in Its Rhizomes
Source: Pharmaceuticals (Basel). 2024 Aug 15;17(8):1074. doi: 10.3390/ph17081074 (PMC11359098; doi:10.3390/ph17081074)
Supplement: Supplementary file 1 [file pharmaceuticals-17-01074-s001.zip › Table S1-New and reduced secondary metabolites in three comparison groups.pdf]

Table S1. New and reduced secondary metabolites in the comparison group SD3 vs. CP

Index	Compounds	Class I	Class II	Formula	Level	CAS	SD3a	SD3b	SD3c	CPa	CPb	CPc
mws0160	3,4-Dihydroxybenzoic Acid Ethyl Ester (Protocatechuic acid ethyl ester)	Phenolic acids	Phenolic acids	C9H10O4	3	3943-89-3	1.80E+04	2.47E+04	1.46E+04	0.00E+00	0.00E+00	0.00E+00
pma6298	3-Hydroxypyridine	Alkaloids	Pyridine alkaloids	C5H5NO	3	109-00-2	4.35E+05	6.74E+05	4.07E+05	0.00E+00	0.00E+00	0.00E+00
Lmsn003111	1-O-p-Coumaroyl-β-D-glucose*	Phenolic acids	Phenolic acids	C15H18O8	2	7139-64-2	3.83E+04	9.18E+04	5.48E+04	0.00E+00	0.00E+00	0.00E+00
pmp001073	Synephrine; 4-[1-Hydroxy-2-(methylamino)ethyl]phenol	Alkaloids	Phenolamine	C9H13NO2	3	94-07-5	2.38E+06	2.38E+06	4.03E+06	0.00E+00	0.00E+00	0.00E+00
ML10177402	4-Aminosalicylic acid	Phenolic acids	Phenolic acids	C7H7NO3	3	65-49-6	4.93E+04	5.45E+04	5.82E+04	0.00E+00	0.00E+00	0.00E+00
MWSHC20168	Methyl cumalate	Phenolic acids	Phenolic acids	C7H6O4	1	6018-41-3	6.93E+05	7.85E+05	1.43E+06	0.00E+00	0.00E+00	0.00E+00
Wbmp006077	1,10-dimethoxy-6,6-dimethyl-2,9-di(propan-2-yloxy)-5,6,6a,7-tetrahydro-4H-dibenzo[de,g]quinolin-6-ium	Alkaloids	Alkaloids	C26H36NO4+	1	-	1.49E+05	2.26E+05	3.73E+05	0.00E+00	0.00E+00	0.00E+00
mws0444	3-Aminosalicylic acid	Phenolic acids	Phenolic acids	C7H7NO3	3	570-23-0	1.79E+05	1.82E+05	2.04E+05	0.00E+00	0.00E+00	0.00E+00
Hmdp000626	Polygonatine A	Alkaloids	Alkaloids	C9H11NO2	1	-	1.05E+06	1.81E+06	1.63E+06	0.00E+00	0.00E+00	0.00E+00
mws0885	2,4-Dihydroxybenzoic acid	Phenolic acids	Phenolic acids	C7H6O4	3	89-86-1	1.60E+06	1.61E+06	3.07E+06	0.00E+00	0.00E+00	0.00E+00
pmb2795	4-Methoxycinnamic acid	Phenolic acids	Phenolic acids	C10H10O3	3	830-09-1	7.40E+04	5.57E+04	7.67E+04	0.00E+00	0.00E+00	0.00E+00
pmn001394	2,3,5,4'-Tetrahydroxystilbene-2-O-glucoside	Others	Stilbene	C20H22O9	2	82373-94-2	3.21E+04	4.86E+04	4.26E+04	0.00E+00	0.00E+00	0.00E+00
Zblp007704	densiflorol B	Quinones	PhenAnthraquinones	C15H10O4	3	-	1.79E+05	1.49E+05	1.42E+05	0.00E+00	0.00E+00	0.00E+00
Wcdp005613	1,2-dihydro-1-oxo-β-carboline	Alkaloids	Alkaloids	C11H8N2O	1	-	7.59E+05	4.61E+05	6.35E+05	0.00E+00	0.00E+00	0.00E+00
Lmmn001552	Leonuriside A	Phenolic acids	Phenolic acids	C14H20O9	2	121748-12-7	1.74E+05	1.80E+05	4.93E+05	0.00E+00	0.00E+00	0.00E+00
MWSmce339	2-Methoxycinnamic acid	Phenolic acids	Phenolic acids	C10H10O3	2	6099-03-2	8.59E+04	7.05E+04	6.30E+04	0.00E+00	0.00E+00	0.00E+00
mws0183	3,4-Dihydroxybenzoic acid (Protocatechuic acid)*	Phenolic acids	Phenolic acids	C7H6O4	1	99-50-3	1.94E+06	1.92E+06	3.01E+06	0.00E+00	0.00E+00	0.00E+00
Wbkn004126	Javanicolide C	Terpenoids	Ditepenoids	C26H36O11	3	-	4.38E+04	4.66E+04	6.62E+04	0.00E+00	0.00E+00	0.00E+00
Hmlp008487	Blumenol C	Terpenoids	Monoterpenoids	C13H22O2	2	36151-02-7	3.86E+04	2.18E+04	5.45E+04	0.00E+00	0.00E+00	0.00E+00
mws0894	Glycitin	Flavonoids	Isoflavones	C22H22O10	3	40246-10-4	9.91E+04	5.28E+04	6.21E+04	0.00E+00	0.00E+00	0.00E+00
Jmwn002057	2-(3-β-D-glucopyranosyloxy-4-hydroxyphenyl)ethanol-1-O-β-D-glucopyranoside	Phenolic acids	Phenolic acids	C20H30O13	3	-	3.48E+05	3.73E+05	5.15E+05	0.00E+00	0.00E+00	0.00E+00
pme3200	1-Methylguanidine	Alkaloids	Alkaloids	C2H7N3	3	471-29-4	1.51E+05	1.23E+05	1.33E+05	0.00E+00	0.00E+00	0.00E+00
Lmgn001670	Salicylic acid	Phenolic acids	Phenolic acids	C7H6O3	1	69-72-7	2.47E+05	2.64E+05	3.44E+05	0.00E+00	0.00E+00	0.00E+00
Lmtp003569	(R)-Mandelamide	Alkaloids	Alkaloids	C8H9NO2	2	24008-62-6	1.17E+05	1.23E+05	1.39E+05	0.00E+00	0.00E+00	0.00E+00
Wcdp004275	Lauramine oxide	Others	Others	C14H31NO	1	1643-20-5	2.80E+04	2.69E+04	3.92E+04	0.00E+00	0.00E+00	0.00E+00
Wchn005575	1-(4'-Hydroxy-3'-methoxyphenyl)-2-[4''-(3-hydroxypropyl)-2'',6''-dimethoxyphenyl]-propane-1,3-Diol	Others	Others	C21H28O8	2	-	9.91E+04	9.29E+04	1.57E+05	0.00E+00	0.00E+00	0.00E+00
pme0428	3,4,5-Trimethoxycinnamic acid	Phenolic acids	Phenolic acids	C12H14O5	3	90-50-6	1.44E+04	1.06E+04	2.17E+04	0.00E+00	0.00E+00	0.00E+00
MWSHY0163	Aromadendrin (Dihydrokaempferol)	Flavonoids	Flavanonols	C15H12O6	2	480-20-6	1.50E+04	1.57E+04	1.89E+04	0.00E+00	0.00E+00	0.00E+00
pmb3056	Homovanilloylquinic acid	Phenolic acids	Phenolic acids	C16H20O9	3	-	8.39E+03	7.64E+03	1.47E+04	0.00E+00	0.00E+00	0.00E+00
Ladn005852	Graminone A	Lignans and Coumarins	Lignans	C20H20O7	3	161407-72-3	2.93E+04	2.19E+04	3.77E+04	0.00E+00	0.00E+00	0.00E+00
Wbmp011422	5,8-Epidioxyergosta-6,22-dien-3-ol (Ergosterol peroxide)	Steroids	Steroid	C28H44O3	2	2061-64-5	1.85E+04	4.84E+04	2.55E+04	0.00E+00	0.00E+00	0.00E+00
pmp001187	1-(Dihydroxyphenyl)-N2,N3-bis(4-hydroxyphenethyl)-(5-8)-dimethoxy-1,2dihydronaphthalene-2,3-dicarboxamide	Alkaloids	Phenolamine	C36H36N2O8	1	-	4.25E+04	2.29E+04	4.37E+04	0.00E+00	0.00E+00	0.00E+00
MWS1839	Ethylparaben	Phenolic acids	Phenolic acids	C9H10O3	3	120-47-8	2.60E+04	2.70E+04	3.75E+04	0.00E+00	0.00E+00	0.00E+00
Lmmn004685	Nectandrin B	Lignans and Coumarins	Lignans	C20H24O5	2	74683-16-2	1.63E+04	3.58E+04	2.23E+04	0.00E+00	0.00E+00	0.00E+00
Wmlp000056	3,5-Dimethoxy-4-hydroxybenzaldehyde	Others	Aldehyde compounds	C9H10O4	3	-	9.79E+04	9.74E+04	8.19E+04	0.00E+00	0.00E+00	0.00E+00
pmn001399	2,4,6,4'-Tetrahydroxy-stilbene-2-O-glucoside	Others	Stilbene	C20H22O9	2	-	2.14E+04	2.40E+04	2.50E+04	0.00E+00	0.00E+00	0.00E+00
Zmyn002856	3-(Hydroxymethyl)phenol	Phenolic acids	Phenolic acids	C7H8O2	2	620-24-6	7.57E+04	9.11E+04	9.67E+04	0.00E+00	0.00E+00	0.00E+00
pmp000608	Aurantio-obtusin-6-O-Glucoside	Quinones	Anthraquinone	C23H24O12	3	129025-96-3	2.32E+05	2.24E+05	7.43E+05	0.00E+00	0.00E+00	0.00E+00
Xmsn004568	Mudanpioside F	Terpenoids	Monoterpenoids	C16H24O8	1	-	2.86E+04	3.71E+04	6.03E+04	0.00E+00	0.00E+00	0.00E+00
MWSslk186	8-O-Acetylharpagide	Terpenoids	Monoterpenoids	C17H26O11	2	6926-14-3	1.20E+04	9.80E+03	8.70E+03	0.00E+00	0.00E+00	0.00E+00
Wcdp000839	5-methylfurfural	Others	Others	C6H6O2	2	620-02-0	4.17E+05	3.19E+05	4.49E+05	0.00E+00	0.00E+00	0.00E+00
pmb4777	4-Hydroxy-7-methoxycoumarin-β-rhamnoside	Lignans and Coumarins	Coumarins	C16H18O8	2	-	3.74E+03	1.10E+04	1.55E+04	0.00E+00	0.00E+00	0.00E+00
Zmsp000758	1-(Hydroxymethyl)hexahydro-1h-pyrrolizin-2-ol	Alkaloids	Pyrrole alkaloids	C8H15NO2	2	-	1.68E+07	1.88E+07	1.49E+07	0.00E+00	0.00E+00	0.00E+00
Lmwp102713	(S)-Peucedanol	Lignans and Coumarins	Coumarins	C14H16O5	2	-	7.26E+04	5.91E+04	6.95E+04	0.00E+00	0.00E+00	0.00E+00
Wchn003594	4-hydroxy-4-(2-hydroxyphenyl)butanoic acid	Others	Others	C10H12O4	2	-	2.04E+04	2.23E+04	3.88E+04	0.00E+00	0.00E+00	0.00E+00
NK10253223	2-Amino-3-methoxybenzoic acid	Phenolic acids	Phenolic acids	C8H9NO3	3	3177-80-8	3.09E+05	2.70E+05	3.00E+05	0.00E+00	0.00E+00	0.00E+00
mws0025	Pyrogallol	Phenolic acids	Phenolic acids	C6H6O3	3	87-66-1	2.40E+05	2.86E+05	2.58E+05	0.00E+00	0.00E+00	0.00E+00
Lmsn002288	1-O-Caffeoyl-(6-O-glucosyl)-β-D-glucose	Phenolic acids	Phenolic acids	C21H28O14	2	-	2.03E+05	2.27E+05	1.46E+05	0.00E+00	0.00E+00	0.00E+00
MWS5206	4-Hydroxyphenyllactic Acid	Phenolic acids	Phenolic acids	C9H10O4	3	306-23-0	3.29E+04	3.06E+04	4.02E+04	0.00E+00	0.00E+00	0.00E+00
Zmjn003003	3,4-Dimethoxybenzoic acid; Veratric acid	Phenolic acids	Phenolic acids	C9H10O4	3	93-07-2	2.28E+04	3.61E+04	4.63E+04	0.00E+00	0.00E+00	0.00E+00
Hmdp003403	Spirost-5-ene-3,27-diol-27-O-glucoside-3-O-[rhamnosyl(1→4)]glucoside (Polygonatoside D)*	Steroids	Steroidal saponins	C45H72O18	2	-	2.04E+05	5.06E+05	4.27E+05	0.00E+00	0.00E+00	0.00E+00
Yamn004462	6-((4-(3-hydroxy-2-(4-(3-hydroxypropyl)-2-methylphenoxy)propoxy)-2-methoxyphenoxy)methyl)tetrahydro-2H-pyran-2,3,4,5-tetraol	Lignans and Coumarins	Lignans	C26H36O11	1	-	3.15E+04	4.06E+04	5.67E+04	0.00E+00	0.00E+00	0.00E+00
Lmmp002013	Dihydroferuloylputrescine	Alkaloids	Phenolamine	C14H22N2O3	2	-	1.55E+05	1.22E+05	2.21E+05	0.00E+00	0.00E+00	0.00E+00
MWSslk183	7,8-Dihydroxy-4-phenylcoumarin	Lignans and Coumarins	Coumarins	C15H10O4	2	842-01-3	6.72E+05	4.96E+05	5.23E+05	0.00E+00	0.00E+00	0.00E+00
Zmpp000906	Norepinephrine	Alkaloids	Alkaloids	C8H11NO3	1	51-41-2	2.72E+04	1.56E+04	2.33E+04	0.00E+00	0.00E+00	0.00E+00
HJAP050	N-Feruloyl-3-methoxytyramine*	Alkaloids	Phenolamine	C19H21NO5	3	78510-19-7	3.47E+04	3.06E+04	4.82E+04	0.00E+00	0.00E+00	0.00E+00
ZbWp001556	5-guanidino-2-(2-hydroxyethyl)pentanoic acid	Others	Others	C8H17N3O3	2	-	1.23E+05	1.62E+05	1.29E+05	0.00E+00	0.00E+00	0.00E+00
Wayp004564	4-hydroxy-4-(3-pyridyl)-butanoic acid	Alkaloids	Pyridine alkaloids	C9H11NO3	2	15569-97-8	4.23E+05	6.60E+05	3.74E+05	0.00E+00	0.00E+00	0.00E+00
Zbsn003878	Vicenin-3	Flavonoids	Flavones	C26H28O14	2	59914-91-9	3.36E+05	3.78E+05	7.30E+05	0.00E+00	0.00E+00	0.00E+00
Hmsn002272	Demethyl coniferin	Phenolic acids	Phenolic acids	C15H20O8	3	-	7.58E+05	8.61E+05	1.00E+06	0.00E+00	0.00E+00	0.00E+00
Lmyn002788	Methyl gallate	Phenolic acids	Phenolic acids	C8H8O5	3	99-24-1	7.38E+04	6.72E+04	1.07E+05	0.00E+00	0.00E+00	0.00E+00
Wayn000370	N-(1-Deoxy-1-fructosyl)Piperidic acid	Alkaloids	Alkaloids	C10H19NO7	3	-	4.03E+04	4.40E+04	4.58E+04	0.00E+00	0.00E+00	0.00E+00
HJN055	Dihydrocharcone-4'-O-glucoside	Flavonoids	Chalcones	C21H24O10	3	-	1.65E+04	1.89E+04	3.78E+04	0.00E+00	0.00E+00	0.00E+00
MWS20177	5-hydroxy-anthraquinone-2-carboxylic acid	Quinones	Anthraquinone	C15H8O5	2	-	3.42E+04	3.71E+04	3.80E+04	0.00E+00	0.00E+00	0.00E+00
Ladp002110	4-hydroxy-2-oxo-1,2-dihydroquinoline-3-carboxylic acid	Alkaloids	Quinoline alkaloids	C10H7NO4	2	73776-24-6	6.39E+05	4.86E+05	6.22E+05	0.00E+00	0.00E+00	0.00E+00
Lmjp005170	4,9-Dihydroxy-α-Lapachone	Quinones	Quinones	C15H14O5	2	56473-67-7	2.49E+04	1.71E+04	6.39E+04	0.00E+00	0.00E+00	0.00E+00
MA10107783	3-[(1-Carboxyvinyl)oxy]benzoic acid	Phenolic acids	Phenolic acids	C10H8O5	3	16929-37-6	7.03E+04	4.88E+04	8.89E+04	0.00E+00	0.00E+00	0.00E+00
Lmmp003517	Solatuberenol A	Others	Others	C13H20O3	3	-	5.40E+04	3.58E+04	9.45E+04	0.00E+00	0.00E+00	0.00E+00
MWSHC2029	Harman-3-carboxylic acid	Alkaloids	Plumerane	C13H10N2O2	2	22329-38-0	1.08E+05	7.25E+04	1.05E+05	0.00E+00	0.00E+00	0.00E+00
MWSmce518	5,7-Dihydroxy-4-Phenylcoumarin	Lignans and Coumarins	Coumarins	C15H10O4	2	7758-73-8	5.19E+03	5.04E+03	1.22E+04	0.00E+00	0.00E+00	0.00E+00
MWSmce460	2-Piperidone	Alkaloids	Piperidine alkaloids	C5H9NO	2	675-20-7	3.72E+05	2.92E+05	4.67E+05	0.00E+00	0.00E+00	0.00E+00
MWSmce455	Vanillic acid methyl ester	Phenolic acids	Phenolic acids	C9H10O4	3	3943-74-6	2.60E+04	1.96E+04	1.43E+04	0.00E+00	0.00E+00	0.00E+00
mws0982	1-Methylhistamine	Alkaloids	Alkaloids	C6H11N3	2	501-75-7	4.27E+05	3.53E+05	5.89E+05	0.00E+00	0.00E+00	0.00E+00
HJAP051	3-(3,4,5-Trimethoxyphenyl)propan-1-ol	Phenolic acids	Phenolic acids	C12H18O4	2	-	6.39E+04	6.30E+04	6.55E+04	0.00E+00	0.00E+00	0.00E+00
MWSmce208	Norharmane; Beta-Carboline	Alkaloids	Plumerane	C11H8N2	2	244-63-3	4.98E+06	2.79E+06	5.26E+06	0.00E+00	0.00E+00	0.00E+00
Hmln000873	2-O-Galloyl-D-glucose	Phenolic acids	Phenolic acids	C13H16O10	3	-	4.24E+03	1.51E+04	1.88E+04	0.00E+00	0.00E+00	0.00E+00
pme1137	6-Hydroxynicotinic acid	Alkaloids	Pyridine alkaloids	C6H5NO3	2	5006-66-6	4.61E+06	4.99E+06	4.76E+06	0.00E+00	0.00E+00	0.00E+00
Zbdn007423	6,7-Dimethoxy-4-chromanone	Flavonoids	Other Flavonoids	C11H12O4	3	-	1.67E+03	3.65E+03	8.46E+03	0.00E+00	0.00E+00	0.00E+00
MWS2069	2-Methoxy-4-methylphenol	Phenolic acids	Phenolic acids	C8H10O2	3	93-51-6	5.52E+04	3.90E+04	2.92E+04	0.00E+00	0.00E+00	0.00E+00
Xmqp004074	Peucedanol 3'-O-glucoside	Lignans and Coumarins	Coumarins	C20H26O10	2	65891-61-4	2.36E+05	1.67E+05	2.93E+05	0.00E+00	0.00E+00	0.00E+00
Lmmp001410	Dihydrocaffeoylputrescine	Alkaloids	Phenolamine	C13H20N2O3	2	-	1.37E+06	1.57E+06	1.20E+06	0.00E+00	0.00E+00	0.00E+00
Wbjp002870	1,3-Dimethyl-2-oxo-5-methoxyindoline-3-acetamide	Alkaloids	Alkaloids	C13H16N2O3	2	-	4.41E+04	6.99E+04	4.44E+04	0.00E+00	0.00E+00	0.00E+00
pmp001004	Perlolyrine	Alkaloids	Plumerane	C16H12N2O2	1	29700-20-7	5.90E+06	3.39E+06	6.42E+06	0.00E+00	0.00E+00	0.00E+00
pmp001265	2-Amino-4-dihydroxy octadecyl galactoside	Alkaloids	Alkaloids	C24H49NO8	2	-	8.39E+04	1.01E+05	1.10E+05	0.00E+00	0.00E+00	0.00E+00
Hmdp003423	Spirost-5-en-3-ol-3-O-glucosyl(1→2)glucosyl(l→4)galactoside (Neosibiricoside D)*	Steroids	Steroidal saponins	C45H72O18	2	880152-67-0	2.29E+05	4.95E+05	4.53E+05	0.00E+00	0.00E+00	0.00E+00
Lmyn011818	3,19-Epoxy-3,22-dihydroxydammara-20,24-dien-26-oic acid δ-lactone (Semialactone)	Terpenoids	Triterpene	C30H44O4	3	366450-46-6	2.98E+04	2.44E+04	3.20E+04	0.00E+00	0.00E+00	0.00E+00
Zmxp005324	Dihydrodehydrodiconiferyl alcohol	Lignans and Coumarins	Lignans	C20H24O6	3	126253-41-6	2.10E+04	2.28E+04	2.52E+04	0.00E+00	0.00E+00	0.00E+00
Lmhp002153	N-p-Coumaroylhydroxyputrescine	Alkaloids	Phenolamine	C13H18N2O3	2	-	9.34E+04	1.43E+05	7.70E+04	0.00E+00	0.00E+00	0.00E+00
zjgp122326	Pyridine-4-formyl-O-β-D-glucopyranoside	Others	Others	C12H15NO7	2	-	1.77E+05	2.29E+05	2.81E+05	0.00E+00	0.00E+00	0.00E+00
Wcfp006751	Plumbagin; 5-Hydroxy-2-Methyl-1,4-Naphthoquinone	Quinones	Quinones	C11H8O3	2	481-42-5	2.17E+04	2.23E+04	5.12E+04	0.00E+00	0.00E+00	0.00E+00
MWSmce355	Methyl Syringate	Phenolic acids	Phenolic acids	C10H12O5	3	884-35-5	2.42E+04	1.55E+04	1.87E+04	0.00E+00	0.00E+00	0.00E+00
Lhmp110110	Pyramidatin F(iso-01)	Others	Others	C22H24O7	2	-	2.67E+04	2.87E+04	2.27E+04	0.00E+00	0.00E+00	0.00E+00
Wbjp006749	N-(4-Hydroxyphenethyl)-2-(3-methoxy-4-hydroxyphenyl)-5-[(Z)-2-[2-methoxy-4-[(E)-2-[(4-hydroxyphenethyl)carbamoyl]ethenyl]phenoxy]-2-[(4-hydroxyphenethyl)carbamoyl]ethenyl]-7-methoxy-2,3-dihydrobenzofuran-3-carboxamide	Alkaloids	Alkaloids	C54H53N3O12	2	-	1.31E+04	1.02E+04	7.72E+03	0.00E+00	0.00E+00	0.00E+00
mws0098	Indole-2-carboxylic acid	Alkaloids	Plumerane	C9H7NO2	3	1477-50-5	7.01E+04	6.84E+04	6.71E+04	0.00E+00	0.00E+00	0.00E+00
Wbkp005691	3-(4-methoxy-9H-pyrido[3,4-b]indol-1-yl)propanoic acid	Alkaloids	Alkaloids	C15H14N2O3	1	-	6.18E+05	6.37E+05	7.49E+05	0.00E+00	0.00E+00	0.00E+00
Ymsp002308	4'-O-Methylnorbelladine	Alkaloids	Alkaloids	C16H19NO3	2	4579-60-6	7.60E+04	3.81E+04	1.19E+05	0.00E+00	0.00E+00	0.00E+00
MWS1854	4-Aminophenol	Alkaloids	Phenolamine	C6H7NO	2	123-30-8	5.62E+04	4.62E+04	8.66E+04	0.00E+00	0.00E+00	0.00E+00
MWStz201	β-Carboline-1-propanoic acid	Alkaloids	Plumerane	C14H12N2O2	3	89915-39-9	4.90E+05	3.81E+05	3.24E+05	0.00E+00	0.00E+00	0.00E+00
Cmlp002704	Folicanthine	Alkaloids	Plumerane	C24H30N4	3	6879-55-6	2.12E+05	2.57E+05	1.95E+05	0.00E+00	0.00E+00	0.00E+00
pmb0752	3-O-Feruloylquinic acid*	Phenolic acids	Phenolic acids	C17H20O9	3	1899-29-2	1.36E+06	1.26E+06	2.33E+06	0.00E+00	0.00E+00	0.00E+00
mws0005	Tryptamine	Alkaloids	Plumerane	C10H12N2	3	61-54-1	3.16E+05	3.16E+05	3.42E+05	0.00E+00	0.00E+00	0.00E+00
pmp001311	6-Hydroxykaempferol-7,6-O-Diglucoside	Flavonoids	Flavonols	C27H30O17	3	-	1.00E+05	1.53E+05	7.16E+04	0.00E+00	0.00E+00	0.00E+00
MWSmce713	3-(4-Hydroxyphenyl)-1-propanol	Phenolic acids	Phenolic acids	C9H12O2	3	10210-17-0	1.89E+04	1.18E+04	2.19E+04	0.00E+00	0.00E+00	0.00E+00
Sadp008019	5-formyl-2,7-dihydroxy-1,6-dimethyl-9,10-dihydrophenanthrene	Terpenoids	Ditepenoids	C17H16O3	2	-	8.45E+04	6.18E+04	1.01E+05	0.00E+00	0.00E+00	0.00E+00
Qatp004621	Machilin H(ISO1)	Lignans and Coumarins	Lignans	C21H26O6	2	-	1.72E+05	2.23E+05	3.24E+05	0.00E+00	0.00E+00	0.00E+00
Zmpn003044	7-Hydroxycoumarin;Umbelliferone	Lignans and Coumarins	Coumarins	C9H6O3	3	93-35-6	7.83E+03	7.29E+03	8.09E+03	0.00E+00	0.00E+00	0.00E+00
Wadn003423	Salvianic acid C	Phenolic acids	Phenolic acids	C18H18O9	2	-	9.25E+04	9.54E+04	1.56E+05	0.00E+00	0.00E+00	0.00E+00
Cmcp002990	Lochrovidine	Alkaloids	Alkaloids	C22H26N2O4	2	1359-09-7	7.59E+05	7.72E+05	1.59E+06	0.00E+00	0.00E+00	0.00E+00
Hmtn001288	Methyl 2,4-dihydroxyphenylacetate	Phenolic acids	Phenolic acids	C9H10O4	3	67828-42-6	3.80E+04	2.45E+04	4.59E+04	0.00E+00	0.00E+00	0.00E+00
Wmgp004939	N-Desmethoxyrankinidine	Alkaloids	Plumerane	C19H22O2N2	3	-	1.61E+05	1.43E+05	1.62E+05	0.00E+00	0.00E+00	0.00E+00
MW0133705	4'-Methoxyflavone	Flavonoids	Flavones	C16H12O3	3	4143-74-2	5.47E+03	3.98E+03	6.97E+03	0.00E+00	0.00E+00	0.00E+00
Smbp002161	8-ethylnorlobelol	Alkaloids	Piperidine alkaloids	C9H19NO	2	-	6.73E+04	4.64E+04	6.68E+04	0.00E+00	0.00E+00	0.00E+00
Latp004059	4-(Trans)-acetul-3,6,8-trihydroxy-3-methyl-dihydronapht halenone	Others	Chromone	C13H14O5	2	-	1.49E+05	1.79E+05	2.83E+05	0.00E+00	0.00E+00	0.00E+00
Lahp005848	pithecellobiumol B	Others	Chromone	C10H10O4	3	-	1.64E+06	2.26E+06	2.09E+06	0.00E+00	0.00E+00	0.00E+00
Ymjm000116	6-O-Acetylarbutin	Phenolic acids	Phenolic acids	C14H18O8	2	10338-88-2	1.13E+05	1.73E+05	2.68E+05	0.00E+00	0.00E+00	0.00E+00
HJAP048	2,4,5-Trimethoxybenzoic acid	Phenolic acids	Phenolic acids	C10H12O5	2	-	1.15E+06	8.69E+05	9.38E+05	0.00E+00	0.00E+00	0.00E+00
mws1014	Fraxetin (7,8-Dihydroxy-6-methoxycoumarin)	Lignans and Coumarins	Coumarins	C10H8O5	3	574-84-5	1.88E+05	4.43E+05	1.38E+05	0.00E+00	0.00E+00	0.00E+00
Wbhp008068	(1R,4aS,4bR,7R,8R,8aR,9S,10aR)-7-ethenyl-8,8a,9-trihydroxy-1,4a,7-trimethyl-2,3,4,4b,5,6,8,9,10,10a-decahydrophenanthrene-1-carboxylic acid	Terpenoids	Terpene	C20H32O5	3	-	1.53E+05	2.02E+05	3.36E+05	0.00E+00	0.00E+00	0.00E+00
Hmcp001579	Limocitrin-3-O-sophoroside*	Flavonoids	Flavonols	C29H34O18	3	-	2.84E+05	2.03E+05	3.39E+05	0.00E+00	0.00E+00	0.00E+00
Lskp211278	Oxypinnatanine A	Alkaloids	Alkaloids	C10H16N2O5	2	-	1.42E+05	6.23E+04	1.38E+05	0.00E+00	0.00E+00	0.00E+00
pmb2601	7-Hydroxycoumarin-O-rhamnoside	Lignans and Coumarins	Coumarins	C15H16O7	3	-	4.44E+04	5.12E+04	8.74E+04	0.00E+00	0.00E+00	0.00E+00
Lmcp002933	Pilosine	Alkaloids	Alkaloids	C16H18N2O3	1	13640-28-3	5.31E+05	4.05E+05	4.54E+05	0.00E+00	0.00E+00	0.00E+00
Wbtp005739	Dioxyxanthon	Flavonoids	Other Flavonoids	C13H8O4	2	-	3.86E+04	6.82E+04	5.29E+04	0.00E+00	0.00E+00	0.00E+00
pmn001505	Oleanolic acid-3-O-xylosyl(1→3)glucuronide	Terpenoids	Triterpene Saponin	C41H64O13	3	-	1.19E+04	7.80E+03	2.05E+04	0.00E+00	0.00E+00	0.00E+00
Cmxn004016	Dihydromarein	Flavonoids	Chalcones	C21H24O11	3	-	8.32E+03	7.68E+03	1.96E+04	0.00E+00	0.00E+00	0.00E+00
mws1574	Geniposidic acid*	Terpenoids	Monoterpenoids	C16H22O10	3	27741-01-1	3.96E+04	2.87E+04	6.12E+04	0.00E+00	0.00E+00	0.00E+00
Lmtp002942	Apigenin-6,8-di-C-arabinoside*	Flavonoids	Flavones	C25H26O13	1	-	1.45E+06	9.47E+05	1.69E+06	0.00E+00	0.00E+00	0.00E+00
Zmhn002227	4-O-Glucosyl-sinapate	Phenolic acids	Phenolic acids	C17H22O10	1	-	4.86E+04	5.06E+04	4.09E+04	0.00E+00	0.00E+00	0.00E+00
Lmwp003442	Amoenin	Flavonoids	Flavonols	C21H20O11	3	-	8.99E+04	1.23E+05	1.26E+05	0.00E+00	0.00E+00	0.00E+00
Lahp004442	3,4-Dihydro-4-(4'-hydroxyphenyl)-5,7-dihydroxycoumarin	Lignans and Coumarins	Coumarins	C15H12O5	3	-	3.54E+03	7.33E+03	9.16E+03	0.00E+00	0.00E+00	0.00E+00
Lmtp002474	Apigenin-6-C-(2''-glucosyl)arabinoside	Flavonoids	Flavones	C26H28O14	1	-	4.29E+05	4.98E+05	6.85E+05	0.00E+00	0.00E+00	0.00E+00
MWSHC20105	4-ketopinoresinol	Lignans and Coumarins	Lignans	C20H20O7	1	66288-89-9	2.32E+04	3.13E+04	3.24E+04	0.00E+00	0.00E+00	0.00E+00
Yamp002097	1-[2-(Furan-2-yl)-2-oxoethyl]pyrrolidin-2-One	Alkaloids	Alkaloids	C10H11NO3	3	-	4.08E+05	5.12E+05	5.06E+05	0.00E+00	0.00E+00	0.00E+00
mws5028	2-Benzoxazolinone	Alkaloids	Alkaloids	C7H5NO2	2	59-49-4	8.38E+04	1.87E+05	8.05E+04	0.00E+00	0.00E+00	0.00E+00
pmp001189	(5-8)-Hydroxy-1-(hydroxyldimethoxyphenyl)-N2,N3-bis(4-hydroxyphenethyl)-(5-8)-dimethoxy-1,2-dihydronaphthalene-2,3-dicarboxamide	Alkaloids	Phenolamine	C38H40N2O10	2	-	7.64E+03	5.89E+03	8.54E+03	0.00E+00	0.00E+00	0.00E+00
MWSmce632	Vanillin acetate	Phenolic acids	Phenolic acids	C10H10O4	3	881-68-5	8.60E+03	5.52E+03	1.31E+04	0.00E+00	0.00E+00	0.00E+00
MWS3020	N-Acetylcadaverine	Alkaloids	Alkaloids	C7H16N2O	3	32343-73-0	2.00E+05	2.06E+05	2.54E+05	0.00E+00	0.00E+00	0.00E+00
Lmdn001501	Gardoside*	Terpenoids	Monoterpenoids	C16H22O10	3	54835-76-6	2.69E+04	2.94E+04	5.36E+04	0.00E+00	0.00E+00	0.00E+00
Wbjp003603	3-Hydroxy-3-[(6-methyl-2-pyridyl)methyl]-5-methoxyindoline-2-one	Alkaloids	Alkaloids	C16H16N2O3	2	-	4.47E+05	3.83E+05	5.03E+05	0.00E+00	0.00E+00	0.00E+00
Lmtp002822	Apigenin-6-C-arabinoside-8-C-xyloside*	Flavonoids	Flavones	C25H26O13	1	-	1.18E+06	9.85E+05	2.03E+06	0.00E+00	0.00E+00	0.00E+00
Hmdp001950	2',7-Dihydroxy-3',4'-dimethoxyisoflavan	Flavonoids	Other Flavonoids	C17H18O5	2	52250-35-8	1.98E+07	1.09E+07	1.85E+07	0.00E+00	0.00E+00	0.00E+00
pmb3074	5-O-p-Coumaroylquinic acid	Phenolic acids	Phenolic acids	C16H18O8	3	1899-30-5	6.09E+04	9.72E+04	1.72E+05	0.00E+00	0.00E+00	0.00E+00
mws1429	Vomifoliol (Blumenol A)	Terpenoids	Monoterpenoids	C13H20O3	2	189351-15-3	9.84E+03	6.89E+03	8.86E+03	0.00E+00	0.00E+00	0.00E+00
MWSslk011	2-Methoxy-1,4-naphthoquinone (Lawsone methyl ether)	Quinones	Quinones	C11H8O3	2	2348-82-5	2.20E+04	1.63E+04	3.78E+04	0.00E+00	0.00E+00	0.00E+00
Wchn003984	1,4,8-Trihydroxynaphthalene-1-O-glucoside	Quinones	Naphthol	C16H18O8	3	39015-63-9	3.84E+04	3.51E+04	1.54E+05	0.00E+00	0.00E+00	0.00E+00
Zjcp102033	nuatigenin-Glc-Glc-Glc	Steroids	Steroidal saponins	C45H72O19	3	-	7.48E+04	1.39E+05	2.21E+04	0.00E+00	0.00E+00	0.00E+00
Lhfp110832	Furanoeremophilane	Terpenoids	Sesquiterpenoids	C15H22O	3	6750-13-6	5.36E+04	4.79E+04	7.26E+04	0.00E+00	0.00E+00	0.00E+00
Lmzp004885	Tricin (5,7,4'-Trihydroxy-3',5'-dimethoxyflavone)	Flavonoids	Flavones	C17H14O7	2	520-32-1	3.75E+04	1.72E+04	4.13E+04	0.00E+00	0.00E+00	0.00E+00
Lxmp122302	Dehydroxyampelopsisionoside	Terpenoids	Sesquiterpenoids	C19H32O7	3	-	7.41E+04	6.56E+04	6.04E+04	0.00E+00	0.00E+00	0.00E+00
Zmhp102730	6-Hydroxykaempferol-3,6-O-Diglucoside*	Flavonoids	Flavonols	C27H30O17	3	-	1.05E+05	1.49E+05	6.85E+04	0.00E+00	0.00E+00	0.00E+00
Hmcp001628	Limocitrin-3,7-di-O-glucoside*	Flavonoids	Flavonols	C29H34O18	3	-	2.99E+05	2.02E+05	3.37E+05	0.00E+00	0.00E+00	0.00E+00
MWS3149	3-Methoxybenzoic acid	Phenolic acids	Phenolic acids	C8H8O3	3	586-38-9	5.47E+03	5.08E+03	3.20E+03	0.00E+00	0.00E+00	0.00E+00
MWS20194	Cinnamic acid	Phenolic acids	Phenolic acids	C9H8O2	3	140-10-3	7.86E+05	3.43E+05	1.06E+06	0.00E+00	0.00E+00	0.00E+00
Layp003558	3,4,5-Trimethoxyphenol-1-O-β-D-glucoside	Phenolic acids	Phenolic acids	C15H22O9	2	-	1.75E+05	2.59E+05	3.25E+05	0.00E+00	0.00E+00	0.00E+00
Lazn004665	Triacanthinapioside	Alkaloids	Alkaloids	C15H21N5O4	1	-	8.27E+04	4.63E+04	5.76E+04	0.00E+00	0.00E+00	0.00E+00
Lamp006064	3-[(3,4-dihydroxyphenyl)methylidene]-5,7-dihydroxy-6-methoxy-2h-1-benzopyran-4-one glucosyl rhamnoside	Flavonoids	Other Flavonoids	C29H34O16	1	-	1.52E+06	1.33E+06	3.37E+06	0.00E+00	0.00E+00	0.00E+00
Lmcp005645	Quercetin-3,4'-Dimethyl Ether	Flavonoids	Flavonols	C17H14O7	2	33429-83-3	6.37E+04	5.33E+04	4.56E+04	0.00E+00	0.00E+00	0.00E+00
Lajp005753	5,3'-dihydroxy-6,7,4'-trimethoxyflavone-8-O-β-D-glucoside	Flavonoids	Flavones	C23H24O12	3	-	1.24E+05	2.38E+05	4.75E+05	0.00E+00	0.00E+00	0.00E+00
Smnp002454	Aloin B	Quinones	Anthraquinone	C21H22O9	3	28371-16-6	1.92E+04	1.42E+04	3.52E+04	0.00E+00	0.00E+00	0.00E+00
MWSHY0057	Baicalin	Flavonoids	Flavones	C21H18O11	2	21967-41-9	3.08E+04	3.52E+04	2.35E+04	0.00E+00	0.00E+00	0.00E+00
Hmjp006104	N-Cis-Feruloyl-3'-O-methyldopamine*	Alkaloids	Phenolamine	C19H21NO5	3	-	3.02E+04	1.94E+04	5.22E+04	0.00E+00	0.00E+00	0.00E+00
Lmtp002642	Apigenin-8-C-(2''-glucosyl)arabinoside	Flavonoids	Flavones	C26H28O14	1	-	4.70E+05	4.63E+05	7.70E+05	0.00E+00	0.00E+00	0.00E+00
Lmsn002548	1-O-Caffeoyl-β-D-glucose	Phenolic acids	Phenolic acids	C15H18O9	3	14364-08-0	2.99E+04	1.35E+04	4.58E+04	0.00E+00	0.00E+00	0.00E+00
Zjbp081718	27-Hydroxyspirost-5-en-3-yl-O-rhamnosyl-(1→2)-O-[glucosyl-(1→6)]-glucoside	Steroids	Steroidal saponins	C45H72O18	1	-	2.69E+05	4.09E+05	3.50E+05	0.00E+00	0.00E+00	0.00E+00
Lmmp002796	Gossypetin-3-O-rutinoside	Flavonoids	Flavonols	C27H30O17	3	-	0.00E+00	0.00E+00	0.00E+00	1.73E+06	2.18E+05	2.40E+05
pma0702	N1,N8-Bis(sinapoyl)spermidine	Alkaloids	Phenolamine	C29H39N3O8	3	-	0.00E+00	0.00E+00	0.00E+00	5.33E+04	1.17E+04	5.68E+04
HX1356	(7R,8S)-9-acetyl-dehydrodiconiferyl alcohol	Lignans and Coumarins	Lignans	C22H24O7	3	-	0.00E+00	0.00E+00	0.00E+00	3.15E+04	1.97E+04	2.26E+04

Table S1. New and reduced secondary metabolites in the comparison group SD6 vs. SD3

Index	Compounds	Class I	Class II	Formula	Level	CAS	SD6a	SD6b	SD6c	SD3a	SD3b	SD3c
Lacp001298	4-Hydroxy-3-methoxy-β-phenethylamine	Alkaloids	Phenolamine	C9H13NO2	2	-	2.00E+05	1.65E+05	1.70E+05	0.00E+00	0.00E+00	0.00E+00
mws0014	Ferulic acid*	Phenolic acids	Phenolic acids	C10H10O4	2	537-98-4	9.08E+04	8.81E+04	7.38E+04	0.00E+00	0.00E+00	0.00E+00
MWStz104	3,4-Dimethoxybenzamide	Alkaloids	Phenolamine	C9H11NO3	3	1521-41-1	4.88E+05	3.90E+05	3.04E+05	0.00E+00	0.00E+00	0.00E+00
mws0346	3-(3-Hydroxyphenyl)-propionic acid	Phenolic acids	Phenolic acids	C9H10O3	3	621-54-5	2.94E+04	1.97E+04	2.75E+04	0.00E+00	0.00E+00	0.00E+00
Lajp007517	7-Methoxy-8-(2'-methoxy-3'-hydroxy-3'-methylbutyl)coumarin	Lignans and Coumarins	Coumarins	C16H20O5	2	-	3.08E+04	3.46E+04	1.53E+04	0.00E+00	0.00E+00	0.00E+00
Lmsn003297	3,4,2',4',6'-Pentahydroxychalcone-4'-O-glucoside	Flavonoids	Chalcones	C21H22O11	3	-	2.41E+05	2.37E+05	1.89E+05	0.00E+00	0.00E+00	0.00E+00
Zmdn004719	2-(2'-hydroxypropyl)-5-methyl-7-hydroxychromone	Others	Chromone	C13H14O4	2	-	2.09E+05	1.99E+05	1.47E+05	0.00E+00	0.00E+00	0.00E+00
Salp002057	neoverataline A	Alkaloids	Steroid alkaloids	C27H41NO8	3	-	1.77E+07	2.07E+07	1.38E+07	0.00E+00	0.00E+00	0.00E+00
pmn001581	Sexangularetin	Flavonoids	Flavonols	C16H12O7	3	571-74-4	4.81E+04	4.81E+04	4.75E+04	0.00E+00	0.00E+00	0.00E+00
Wdtp003242	methyl-4,5-diacetyloxy-6-[4-(hydroxymethyl)phenoxy]-3-methyloxane-2-carboxylate	Phenolic acids	Phenolic acids	C19H24O9	2	-	1.57E+05	1.47E+05	1.26E+05	0.00E+00	0.00E+00	0.00E+00
Lasp006911	1,3,8-Trihydroxy-10-methylacridin-9(10H)-one	Alkaloids	Quinorisidine alkaloids	C14H11NO4	3	-	9.93E+04	6.17E+04	7.20E+04	0.00E+00	0.00E+00	0.00E+00
Hmdp001676	N-p-Coumaroyloctopamine	Alkaloids	Phenolamine	C17H17NO4	3	66648-45-1	3.06E+05	3.33E+05	3.03E+05	0.00E+00	0.00E+00	0.00E+00
pma0702	N1,N8-Bis(sinapoyl)spermidine	Alkaloids	Phenolamine	C29H39N3O8	3	-	7.78E+03	5.73E+03	1.01E+04	0.00E+00	0.00E+00	0.00E+00
Wasn003547	Heptane-1,7-diol sulfate	Others	Others	C7H16O5S	3	-	7.07E+04	7.41E+04	5.72E+04	0.00E+00	0.00E+00	0.00E+00
MWSHY0017	Naringenin (5,7,4'-Trihydroxyflavanone)*	Flavonoids	Flavanones	C15H12O5	3	480-41-1	5.12E+04	5.83E+04	5.07E+04	0.00E+00	0.00E+00	0.00E+00
mws0044	Taxifolin(Dihydroquercetin)	Flavonoids	Flavanonols	C15H12O7	3	480-18-2	1.63E+04	1.45E+04	1.01E+04	0.00E+00	0.00E+00	0.00E+00
Wbjp001169	o-Carboxy-5-hydroxytryptamine	Alkaloids	Alkaloids	C11H12N2O3	2	-	0.00E+00	0.00E+00	0.00E+00	1.60E+05	8.38E+04	9.67E+04
Zbfn002690	1-(4-Hydroxybenzoyl)Glucose; 25545-07-7	Phenolic acids	Phenolic acids	C13H16O8	3	-	0.00E+00	0.00E+00	0.00E+00	1.67E+04	1.45E+04	1.34E+04
Zmbp002538	1-Methoxy-indole-3-acetamide	Alkaloids	Plumerane	C11H12N2O2	1	-	0.00E+00	0.00E+00	0.00E+00	5.02E+05	4.22E+05	2.37E+05
MWSmce621	Chicoric Acid	Phenolic acids	Phenolic acids	C22H18O12	3	70831-56-0	0.00E+00	0.00E+00	0.00E+00	4.71E+03	1.65E+04	1.12E+04
Lmhp003217	2'-Hydoxy-5-methoxyGenistein-O-rhamnosyl-glucoside	Flavonoids	Isoflavones	C28H32O16	3	-	0.00E+00	0.00E+00	0.00E+00	7.94E+04	8.91E+04	1.01E+05
Lahp002608	3,5-Dihydro-2H-Furo[3,2-C]Quinolin-4-One	Alkaloids	Quinoline alkaloids	C11H9NO2	1	-	0.00E+00	0.00E+00	0.00E+00	1.32E+06	1.14E+06	6.58E+05
Wagp002530	2-Methoxy-1-benzofuran-5-carbaldehyde	Others	Others	C10H8O3	3	-	0.00E+00	0.00E+00	0.00E+00	3.43E+05	4.17E+05	2.77E+05
pmb0502	p-Coumaroylspermidine	Alkaloids	Phenolamine	C16H25N3O2	2	-	0.00E+00	0.00E+00	0.00E+00	8.32E+05	8.42E+05	5.65E+05
Lahp001956	Pyran[3,4-b]indole-2-ketone	Alkaloids	Plumerane	C11H9O2N	3	-	0.00E+00	0.00E+00	0.00E+00	1.50E+06	1.14E+06	6.22E+05
Hmmp001310	3-Indoleacrylic acid	Alkaloids	Plumerane	C11H9NO2	1	1204-06-4	0.00E+00	0.00E+00	0.00E+00	7.97E+06	5.96E+06	3.12E+06
pmb0818	Methoxyindoleacetic acid	Alkaloids	Plumerane	C11H11NO3	3	3471-31-6	0.00E+00	0.00E+00	0.00E+00	1.13E+06	8.84E+05	4.67E+05
Saln003574	Genameside C	Terpenoids	Monoterpenoids	C23H34O15	3	-	0.00E+00	0.00E+00	0.00E+00	4.34E+04	5.14E+04	4.16E+04
mws0059	Quercetin-3-O-rutinoside (Rutin)	Flavonoids	Flavonols	C27H30O16	3	153-18-4	0.00E+00	0.00E+00	0.00E+00	1.27E+04	1.17E+04	6.38E+03
Lmsn000363	Raspberryketone glucoside	Phenolic acids	Phenolic acids	C16H22O7	3	38963-94-9	0.00E+00	0.00E+00	0.00E+00	3.73E+04	3.50E+04	4.10E+04
Hmjp002999	Isorhamnetin-3-O-rutinoside (Narcissin)*	Flavonoids	Flavonols	C28H32O16	3	604-80-8	0.00E+00	0.00E+00	0.00E+00	7.40E+04	1.67E+04	9.62E+04
Wcjp002598	8-hydroxyquinoline	Alkaloids	Quinoline alkaloids	C9H7NO	2	148-24-3	0.00E+00	0.00E+00	0.00E+00	4.57E+05	4.44E+05	2.06E+05
Lmsp004670	Kaempferol-3-O-glucoside-7-O-rhamnoside*	Flavonoids	Flavonols	C27H30O15	3	2392-95-2	0.00E+00	0.00E+00	0.00E+00	6.88E+03	3.97E+03	1.68E+04
Lazn005175	1-(4-Hydroxy-3,5-dimethoxyphenyl)-2-[4-(3-hydroxypropyl)-2,6-dimethoxyphenoxy]propane-1,3-diol	Lignans and Coumarins	Lignans	C22H30O9	3	-	0.00E+00	0.00E+00	0.00E+00	3.94E+03	6.15E+03	7.66E+03
pmp001311	6-Hydroxykaempferol-7,6-O-Diglucoside	Flavonoids	Flavonols	C27H30O17	3	-	0.00E+00	0.00E+00	0.00E+00	1.00E+05	1.53E+05	7.16E+04
Lmsp004721	Tamarixetin-3-O-glucoside-7-O-rhamnoside*	Flavonoids	Flavonols	C28H32O16	3	-	0.00E+00	0.00E+00	0.00E+00	9.52E+04	2.60E+04	1.74E+05
Wbjp002330	(3S)-1,3-dihydroxy-2,3,3a,4-tetrahydropyrrolo[2,1-b]quinazolin-9(1H)-one	Alkaloids	Alkaloids	C11H12N2O3	2	-	0.00E+00	0.00E+00	0.00E+00	6.66E+05	5.39E+05	3.24E+05
pmb0819	3-Indoleacetonitrile	Alkaloids	Plumerane	C10H8N2	3	771-51-7	0.00E+00	0.00E+00	0.00E+00	1.77E+05	1.41E+05	1.12E+05
Qahn003953	Erigeside B	Others	Others	C12H22O6	3	95632-87-4	0.00E+00	0.00E+00	0.00E+00	3.67E+04	1.44E+04	2.74E+04
Lmmp002463	Sexangularetin-3-O-glucoside-7-O-rhamnoside*	Flavonoids	Flavonols	C28H32O16	3	-	0.00E+00	0.00E+00	0.00E+00	9.30E+04	2.46E+04	1.63E+05
Wabn003563	3'-O-Beta-D-Glucopyranosyl plumbagic acid	Phenolic acids	Phenolic acids	C17H22O10	2	-	0.00E+00	0.00E+00	0.00E+00	5.36E+04	3.78E+04	3.41E+04
Zmhp102730	6-Hydroxykaempferol-3,6-O-Diglucoside*	Flavonoids	Flavonols	C27H30O17	3	-	0.00E+00	0.00E+00	0.00E+00	1.05E+05	1.49E+05	6.85E+04
Lman003972	6-O-Caffeoylarbutin	Phenolic acids	Phenolic acids	C21H22O10	2	136172-60-6	0.00E+00	0.00E+00	0.00E+00	3.49E+05	5.30E+04	1.60E+04
Hagn003556	7-Benzyloxy-5-hydroxy-3',4'-methylenedioxyflavonoid	Flavonoids	Flavones	C23H16O6	2	-	0.00E+00	0.00E+00	0.00E+00	2.64E+04	2.38E+04	4.15E+04
Labn004625	2-Phenylethyl beta-primeveroside	Phenolic acids	Phenolic acids	C19H28O10	1	129932-48-5	0.00E+00	0.00E+00	0.00E+00	2.51E+04	8.86E+03	1.67E+04
pmn001583	Quercetin-3-O-robinobioside	Flavonoids	Flavonols	C27H30O16	3	52525-35-6	0.00E+00	0.00E+00	0.00E+00	1.37E+04	8.76E+03	9.71E+03
Zjzp081836	2-Hydroxy diosgenin-Glc-Glc-Glc	Steroids	Steroidal saponins	C45H72O19	3	-	0.00E+00	0.00E+00	0.00E+00	2.74E+04	9.32E+04	2.65E+04
Wcdn004141	2-[3-[4-[1,3-dihydroxy-1-(4-hydroxy-3-methoxyphenyl)propan-2-yl]oxy-3-methoxyphenyl]propoxy]-6-(hydroxymethyl)oxane-3,4,5-triol	Others	Others	C26H36O12	3	-	0.00E+00	0.00E+00	0.00E+00	1.57E+04	2.45E+04	2.36E+04
MWSHC2041	sophorabioside	Flavonoids	Isoflavones	C27H30O14	3	2945-88-2	0.00E+00	0.00E+00	0.00E+00	7.28E+04	2.70E+04	1.16E+05

Table S1. New and reduced secondary metabolites in the comparison group SD9 vs. SD6

Index	Compounds	Class I	Class II	Formula	Level	CAS	SD9a	SD9b	SD9c	SD6a	SD6b	SD6c
Saln003574	Genameside C	Terpenoids	Monoterpenoids	C23H34O15	3	-	1.93E+04	1.92E+04	2.23E+04	0.00E+00	0.00E+00	0.00E+00
Sazp005659	Comazaphilone D	Others	Others	C21H22O7	3	-	7.47E+03	2.29E+04	1.99E+04	0.00E+00	0.00E+00	0.00E+00
Lman003972	6-O-Caffeoylarbutin	Phenolic acids	Phenolic acids	C21H22O10	2	136172-60-6	9.39E+03	9.40E+03	3.19E+04	0.00E+00	0.00E+00	0.00E+00
Wcdn004141	2-[3-[4-[1,3-dihydroxy-1-(4-hydroxy-3-methoxyphenyl)propan-2-yl]oxy-3-methoxyphenyl]propoxy]-6-(hydroxymethyl)oxane-3,4,5-triol	Others	Others	C26H36O12	3	-	5.83E+03	5.41E+03	1.18E+04	0.00E+00	0.00E+00	0.00E+00
Yacn003968	Cantleyine	Alkaloids	Alkaloids	C11H13NO3	3	30333-81-4	0.00E+00	0.00E+00	0.00E+00	2.95E+03	9.84E+03	7.80E+03
Lmmn001552	Leonuriside A	Phenolic acids	Phenolic acids	C14H20O9	2	121748-12-7	0.00E+00	0.00E+00	0.00E+00	8.99E+03	4.73E+04	3.57E+04
Lmmn001294	Koaburaside*	Phenolic acids	Phenolic acids	C14H20O9	1	41653-73-0	0.00E+00	0.00E+00	0.00E+00	1.34E+04	1.94E+04	2.53E+04
Hmgp002327	3-amino-2-naphthoic acid	Alkaloids	Alkaloids	C11H9NO2	1	-	0.00E+00	0.00E+00	0.00E+00	4.80E+06	2.03E+04	1.09E+04
MWSHY0064	Isorhamnetin-3-O-neohesperidoside*	Flavonoids	Flavonols	C28H32O16	3	55033-90-4	0.00E+00	0.00E+00	0.00E+00	4.11E+03	1.10E+04	2.59E+03
pmp000087	2-Feruloyl-sn-glycerol	Phenolic acids	Phenolic acids	C13H16O6	3	-	0.00E+00	0.00E+00	0.00E+00	1.43E+04	2.09E+04	8.01E+03
Hmtn001302	Glucosyloxybenzoic acid	Phenolic acids	Phenolic acids	C13H16O8	3	-	0.00E+00	0.00E+00	0.00E+00	7.79E+04	9.91E+04	4.21E+04
Lcdn000131	sachaliside	Others	Others	C15H20O7	3	132294-76-9	0.00E+00	0.00E+00	0.00E+00	4.99E+04	2.61E+04	4.86E+04
pme2292	Putrescine	Alkaloids	Alkaloids	C4H12N2	3	110-60-1	0.00E+00	0.00E+00	0.00E+00	1.62E+05	2.40E+05	2.09E+05
pmp001265	2-Amino-4-dihydroxy octadecyl galactoside	Alkaloids	Alkaloids	C24H49NO8	2	-	0.00E+00	0.00E+00	0.00E+00	4.50E+04	3.86E+04	4.90E+04
Zmxp005324	Dihydrodehydrodiconiferyl alcohol	ignans and Coumarin	Lignans	C20H24O6	3	126253-41-6	0.00E+00	0.00E+00	0.00E+00	1.83E+04	2.33E+04	1.95E+04
Zjhp090617	5-hydroxy-3-(2-hydroxy-4-methoxybenzyl)-7-methoxychroman-4-one	Flavonoids	Other Flavonoids	C18H18O6	3	-	0.00E+00	0.00E+00	0.00E+00	6.63E+03	2.21E+04	1.32E+04
pmn001338	Rhein-8-O-glucoside	Quinones	Anthraquinone	C21H18O11	3	34298-86-7	0.00E+00	0.00E+00	0.00E+00	3.32E+03	4.24E+03	6.64E+03
Cmlp002704	Folicanthine	Alkaloids	Plumerane	C24H30N4	3	6879-55-6	0.00E+00	0.00E+00	0.00E+00	1.75E+04	1.84E+04	1.68E+04
Lmzn001582	5'-Glucosyloxyjasmanic acid	Phenolic acids	Phenolic acids	C18H28O9	1	-	0.00E+00	0.00E+00	0.00E+00	3.87E+04	2.74E+04	2.06E+04
Hmhp006830	6,8-Dihydroxy-2-(2-hydroxy-4-methoxybenzyl)-7-methyl-3,4-dihydronaphthalen-1(2H)-one*	Flavonoids	Other Flavonoids	C18H18O6	2	-	0.00E+00	0.00E+00	0.00E+00	1.21E+04	2.94E+04	1.21E+04
Wmgp004939	N-Desmethoxyrankinidine	Alkaloids	Plumerane	C19H22O2N2	3	-	0.00E+00	0.00E+00	0.00E+00	1.38E+04	3.42E+04	5.07E+04
mws0011	Syringin	Phenolic acids	Phenolic acids	C17H24O9	2	118-34-3	0.00E+00	0.00E+00	0.00E+00	7.55E+04	6.98E+04	7.82E+04
Ymjm000116	6-O-Acetylarbutin	Phenolic acids	Phenolic acids	C14H18O8	2	10338-88-2	0.00E+00	0.00E+00	0.00E+00	1.93E+04	1.17E+05	3.64E+04
Saln003605	3'-O-Feruloyl Swertiamarin	Terpenoids	Monoterpenoids	C26H30O13	2	-	0.00E+00	0.00E+00	0.00E+00	1.44E+04	2.30E+04	2.32E+04
pmn001692	3-O-Acetylhamayne	Alkaloids	quinoline alkaloic	C18H19NO5	1	-	0.00E+00	0.00E+00	0.00E+00	2.76E+03	7.30E+03	8.94E+03
Wbwn004173	Heteroclitin A	ignans and Coumarin	Lignans	C28H36O8	3	140369-75-1	0.00E+00	0.00E+00	0.00E+00	1.14E+04	1.43E+04	1.73E+04
Lmyp003408	Orcinol glucoside	Phenolic acids	Phenolic acids	C13H18O7	3	21082-33-7	0.00E+00	0.00E+00	0.00E+00	2.21E+04	3.51E+04	3.33E+04
Wcdn004423	(2S,3R,4R)-3a-[(Beta-D-Glucopyranosyl)Oxy]Lyoniresinol	ignans and Coumarin	Lignans	C28H38O13	1	-	0.00E+00	0.00E+00	0.00E+00	1.04E+04	8.47E+04	4.78E+04
pma0702	N1,N8-Bis(sinapoyl)spermidine	Alkaloids	Phenolamine	C29H39N3O8	3	-	0.00E+00	0.00E+00	0.00E+00	7.78E+03	5.73E+03	1.01E+04
Ymjm000148	Salidroside	Phenolic acids	Phenolic acids	C14H20O7	3	10338-51-9	0.00E+00	0.00E+00	0.00E+00	7.61E+04	5.19E+04	3.57E+04
Layp003558	3,4,5-Trimethoxyphenol-1-O-β-D-glucoside	Phenolic acids	Phenolic acids	C15H22O9	2	-	0.00E+00	0.00E+00	0.00E+00	5.38E+04	2.20E+05	1.18E+05
mws1434	Apigenin-6-C-glucoside (Isovitexin)	Flavonoids	Flavones	C21H20O10	3	38953-85-4	0.00E+00	0.00E+00	0.00E+00	1.64E+04	8.78E+03	1.23E+04
Wbmp003116	10,11-dimethoxy-9-((3,4,5-trihydroxy-6-methyltetrahydro-2H-pyran-2-yl)oxy)-5,6,7a,8-tetrahydro-7H-[1,3]dioxolo[4',5':4,5]benzo[1,2,3-de]benzo[g]quinoline-7-carbaldehyde	Alkaloids	Alkaloids	C26H29NO10	3	-	0.00E+00	0.00E+00	0.00E+00	3.80E+04	7.24E+04	2.21E+04
Lmdn003868	Frehmaglutoside G	Terpenoids	Monoterpenoids	C21H36O9	3	-	0.00E+00	0.00E+00	0.00E+00	1.29E+04	1.49E+04	1.16E+04
