# Supplementary material for: Traditional Processing Can Enhance the Medicinal Effects of Polygonatum cyrtonema by Inducing Significant Chemical Changes in the Functional Components in Its Rhizomes
Source: Pharmaceuticals (Basel). 2024 Aug 15;17(8):1074. doi: 10.3390/ph17081074 (PMC11359098; doi:10.3390/ph17081074)
Supplement: Supplementary file 1 [file pharmaceuticals-17-01074-s001.zip › Table S2-KEGG pathways and annotated compounds in three comparison groups.pdf]

Table S2. KEGG pathways and annotated compounds in the comparison group SD3 vs. CP

Kegg_pathway	ko_ID	Sig_compound	compound	Sig_compound_all	compound_all	IndexList	CIDList	Pathway
Tyrosine metabolism	ko00350	10	11	63	94	mws0180;Lmbn001981;pme1292;pme1002;pme2598;Zmpp000906;MWS4301;mws2368;pme1439;Ymjm000148	C00628+C05585+C00544+C00483+C01161+C00547+C05593+C06044+C00811+C06046	https:\\www.genome.jp\dbget-bin\www_bget?map00350
Metabolic pathways	ko01100	47	66	63	94	mws0180;Lmmn001643;mws2212;pme3083;Lmbn001981;mws0628;mws0183;pme1292;Lmgn001670;MWSHY0163;Lmbn002648;pme1002;pme2598;mws0458;Lmbn002644;mws0467;mws0704;mws0749;MWSmce248;Zmpp000906;pme1841;MWS4301;mws1358;MWSmce118;MWSmce548;MA10107783;pmb0501;Cmmn012997;mws0982;pme1137;Lmbp000728;mws1346;pme2292;MWSslk106;pmb1096;MWSmce466;NK10264324;pmb0764;mws0005;Lmbn002737;mws2368;pmb0819;pme1439;mws4085;Lmrn003000;Ymjm000148;MWS20194	C00628+C01772+C01197+C05653+C05585+C00633+C00230+C00544+C00805+C00974+C02763+C00483+C01161+C00755+C06758+C01744+C00346+C00156+C12621+C00547+C01672+C05593+C00090+C02835+C00719+C20772+C00179+C06087+C05127+C01020+C20782+C00956+C00134+C05332+C00463+C10700+C02183+C04294+C00398+C07209+C06044+C02938+C00811+C00482+C05607+C06046+C00423	https:\\www.genome.jp\dbget-bin\www_bget?map01100
Phenylalanine metabolism	ko00360	8	9	63	94	Lmmn001643;Lmbn002648;MWSmce248;MWS4301;ML10179289;MWSslk106;Lmrn003000;MWS20194	C01772+C02763+C12621+C05593+C05853+C05332+C05607+C00423	https:\\www.genome.jp\dbget-bin\www_bget?map00360
Biosynthesis of various plant secondary metabolites	ko00999	7	8	63	94	Lmmn001643;Zmln002252;pmb0501;pmb1096;Zmpn003044;mws1014;MWS20194	C01772+C22007+C00179+C00463+C09315+C09265+C00423	https:\\www.genome.jp\dbget-bin\www_bget?map00999
Biosynthesis of secondary metabolites	ko01110	28	40	63	94	Lmmn001643;mws2212;mws0639;mws0183;Lmgn001670;MWSHY0163;Zmln002252;pme1002;mws0458;mws0749;pme1841;MA10107783;pmb0501;Cmmn012997;mws1346;pme2292;pmb1096;mws0005;pmp000509;Zmpn003044;mws1014;pme1439;mws1574;mws4085;Lmrn003000;MWSCX015;pmb3074;MWS20194	C01772+C01197+C00196+C00230+C00805+C00974+C22007+C00483+C00755+C00156+C01672+C20772+C00179+C06087+C00956+C00134+C00463+C00398+C11918+C09315+C09265+C00811+C11673+C00482+C05607+C10945+C12208+C00423	https:\\www.genome.jp\dbget-bin\www_bget?map01110
Isoquinoline alkaloid biosynthesis	ko00950	3	3	63	94	Hmgn001653;pme1002;pme1439	C16700+C00483+C00811	https:\\www.genome.jp\dbget-bin\www_bget?map00950
Phenylpropanoid biosynthesis	ko00940	8	11	63	94	mws2212;mws0011;Lhhp120814;pme1439;mws4085;MWSCX015;pmb3074;MWS20194	C01197+C01533+C10453+C00811+C00482+C10945+C12208+C00423	https:\\www.genome.jp\dbget-bin\www_bget?map00940
Tryptophan metabolism	ko00380	7	7	63	94	pme3083;Zmtn001624;NK10253223;pmb0818;pmb1096;mws0005;pmb0819	C05653+C02172+C05831+C05660+C00463+C00398+C02938	https:\\www.genome.jp\dbget-bin\www_bget?map00380
Phenylalanine, tyrosine and tryptophan biosynthesis	ko00400	2	2	63	94	mws0183;pmb1096	C00230+C00463	https:\\www.genome.jp\dbget-bin\www_bget?map00400
Isoflavonoid biosynthesis	ko00943	1	2	63	94	mws0894	C16195	https:\\www.genome.jp\dbget-bin\www_bget?map00943
Ubiquinone and other terpenoid-quinone biosynthesis	ko00130	5	5	63	94	pme1292;mws0749;MA10107783;pme1439;MWS20194	C00544+C00156+C20772+C00811+C00423	https:\\www.genome.jp\dbget-bin\www_bget?map00130
Biosynthesis of cofactors	ko01240	5	6	63	94	pme1292;pme1002;mws0749;MA10107783;pmb0764	C00544+C00483+C00156+C20772+C04294	https:\\www.genome.jp\dbget-bin\www_bget?map01240
Biosynthesis of various alkaloids	ko00996	3	3	63	94	Lmgn001670;mws0458;MWS20194	C00805+C00755+C00423	https:\\www.genome.jp\dbget-bin\www_bget?map00996
Plant hormone signal transduction	ko04075	1	1	63	94	Lmgn001670	C00805	https:\\www.genome.jp\dbget-bin\www_bget?map04075
Flavonoid biosynthesis	ko00941	2	4	63	94	MWSHY0163;pmb3074	C00974+C12208	https:\\www.genome.jp\dbget-bin\www_bget?map00941
Glycosylphosphatidylinositol (GPI)-anchor biosynthesis	ko00563	1	1	63	94	mws0704	C00346	https:\\www.genome.jp\dbget-bin\www_bget?map00563
Glycerophospholipid metabolism	ko00564	1	2	63	94	mws0704	C00346	https:\\www.genome.jp\dbget-bin\www_bget?map00564
Sphingolipid metabolism	ko00600	1	1	63	94	mws0704	C00346	https:\\www.genome.jp\dbget-bin\www_bget?map00600
Folate biosynthesis	ko00790	1	1	63	94	mws0749	C00156	https:\\www.genome.jp\dbget-bin\www_bget?map00790
Lysine degradation	ko00310	2	2	63	94	pme1841;mws1346	C01672+C00956	https:\\www.genome.jp\dbget-bin\www_bget?map00310
D-Amino acid metabolism	ko00470	2	2	63	94	pme1841;pme2292	C01672+C00134	https:\\www.genome.jp\dbget-bin\www_bget?map00470
Glutathione metabolism	ko00480	2	4	63	94	pme1841;pme2292	C01672+C00134	https:\\www.genome.jp\dbget-bin\www_bget?map00480
Tropane, piperidine and pyridine alkaloid biosynthesis	ko00960	3	6	63	94	pme1841;pme2292;Lmrn003000	C01672+C00134+C05607	https:\\www.genome.jp\dbget-bin\www_bget?map00960
Histidine metabolism	ko00340	2	4	63	94	MWSmce118;mws0982	C02835+C05127	https:\\www.genome.jp\dbget-bin\www_bget?map00340
Glycine, serine and threonine metabolism	ko00260	1	1	63	94	MWSmce548	C00719	https:\\www.genome.jp\dbget-bin\www_bget?map00260
ABC transporters	ko02010	2	4	63	94	MWSmce548;pme2292	C00719+C00134	https:\\www.genome.jp\dbget-bin\www_bget?map02010
Arginine and proline metabolism	ko00330	2	7	63	94	pmb0501;pme2292	C00179+C00134	https:\\www.genome.jp\dbget-bin\www_bget?map00330
Diterpenoid biosynthesis	ko00904	1	2	63	94	Cmmn012997	C06087	https:\\www.genome.jp\dbget-bin\www_bget?map00904
Nicotinate and nicotinamide metabolism	ko00760	1	2	63	94	pme1137	C01020	https:\\www.genome.jp\dbget-bin\www_bget?map00760
Lysine biosynthesis	ko00300	1	1	63	94	mws1346	C00956	https:\\www.genome.jp\dbget-bin\www_bget?map00300
2-Oxocarboxylic acid metabolism	ko01210	1	1	63	94	mws1346	C00956	https:\\www.genome.jp\dbget-bin\www_bget?map01210
Biosynthesis of amino acids	ko01230	1	2	63	94	mws1346	C00956	https:\\www.genome.jp\dbget-bin\www_bget?map01230
Benzoxazinoid biosynthesis	ko00402	1	1	63	94	pmb1096	C00463	https:\\www.genome.jp\dbget-bin\www_bget?map00402
Thiamine metabolism	ko00730	1	1	63	94	pmb0764	C04294	https:\\www.genome.jp\dbget-bin\www_bget?map00730
Indole alkaloid biosynthesis	ko00901	1	1	63	94	mws0005	C00398	https:\\www.genome.jp\dbget-bin\www_bget?map00901
Polyketide sugar unit biosynthesis	ko00523	1	1	63	94	pmp000509	C11918	https:\\www.genome.jp\dbget-bin\www_bget?map00523
Monoterpenoid biosynthesis	ko00902	1	1	63	94	mws1574	C11673	https:\\www.genome.jp\dbget-bin\www_bget?map00902
Stilbenoid, diarylheptanoid and gingerol biosynthesis	ko00945	1	1	63	94	pmb3074	C12208	https:\\www.genome.jp\dbget-bin\www_bget?map00945

Table S2. KEGG pathways and annotated compounds in the comparison group SD6 vs. SD3

Kegg_pathway	ko_ID	Sig_compound	compound	Sig_compound_all	compound_all	IndexList	CIDList	Pathway
Phenylpropanoid biosynthesis	ko00940	5	12	34	99	mws2212;HJN003;mws0014;pme1439;mws4085	C01197+C01175+C01494+C00811+C00482	https:\\www.genome.jp\dbget-bin\www_bget?map00940
Metabolic pathways	ko01100	25	70	34	99	mws2212;MWSHY0163;Lmlp003161;mws0704;mws0014;Zmpp000906;pme1841;mws1358;mws0059;pmb0501;Cmmn012997;pmb0490;mws0346;mws0982;pme1137;pme2292;pmb1096;NK10264324;pmb0764;pmb0819;pme1439;mws4085;pme2693;MWSHY0017;mws0044	C01197+C00974+C10497+C00346+C01494+C00547+C01672+C00090+C05625+C00179+C06087+C18326+C11457+C05127+C01020+C00134+C00463+C02183+C04294+C02938+C00811+C00482+C02714+C00509+C01617	https:\\www.genome.jp\dbget-bin\www_bget?map01100
Biosynthesis of secondary metabolites	ko01110	18	44	34	99	mws2212;MWSHY0163;Zmln002252;HJN003;mws0014;pme1841;mws0059;pmb0501;Cmmn012997;Lmsn003297;pme2292;pmb1096;pmp000509;Zmpn003044;pme1439;mws4085;MWSHY0017;mws0044	C01197+C00974+C22007+C01175+C01494+C01672+C05625+C00179+C06087+C16408+C00134+C00463+C11918+C09315+C00811+C00482+C00509+C01617	https:\\www.genome.jp\dbget-bin\www_bget?map01110
Flavonoid biosynthesis	ko00941	5	7	34	99	MWSHY0163;Lmsn003297;Zbsp007084;MWSHY0017;mws0044	C00974+C16408+C09614+C00509+C01617	https:\\www.genome.jp\dbget-bin\www_bget?map00941
Biosynthesis of various plant secondary metabolites	ko00999	4	8	34	99	Zmln002252;pmb0501;pmb1096;Zmpn003044	C22007+C00179+C00463+C09315	https:\\www.genome.jp\dbget-bin\www_bget?map00999
Arginine and proline metabolism	ko00330	5	7	34	99	Lmlp003161;pmb0501;pmb0490;pme2292;pme2693	C10497+C00179+C18326+C00134+C02714	https:\\www.genome.jp\dbget-bin\www_bget?map00330
Glycosylphosphatidylinositol (GPI)-anchor biosynthesis	ko00563	1	1	34	99	mws0704	C00346	https:\\www.genome.jp\dbget-bin\www_bget?map00563
Glycerophospholipid metabolism	ko00564	1	2	34	99	mws0704	C00346	https:\\www.genome.jp\dbget-bin\www_bget?map00564
Sphingolipid metabolism	ko00600	1	1	34	99	mws0704	C00346	https:\\www.genome.jp\dbget-bin\www_bget?map00600
Tyrosine metabolism	ko00350	2	11	34	99	Zmpp000906;pme1439	C00547+C00811	https:\\www.genome.jp\dbget-bin\www_bget?map00350
Lysine degradation	ko00310	1	2	34	99	pme1841	C01672	https:\\www.genome.jp\dbget-bin\www_bget?map00310
D-Amino acid metabolism	ko00470	2	2	34	99	pme1841;pme2292	C01672+C00134	https:\\www.genome.jp\dbget-bin\www_bget?map00470
Glutathione metabolism	ko00480	2	4	34	99	pme1841;pme2292	C01672+C00134	https:\\www.genome.jp\dbget-bin\www_bget?map00480
Tropane, piperidine and pyridine alkaloid biosynthesis	ko00960	2	6	34	99	pme1841;pme2292	C01672+C00134	https:\\www.genome.jp\dbget-bin\www_bget?map00960
Tryptophan metabolism	ko00380	3	7	34	99	pmb0818;pmb1096;pmb0819	C05660+C00463+C02938	https:\\www.genome.jp\dbget-bin\www_bget?map00380
Flavone and flavonol biosynthesis	ko00944	3	5	34	99	mws0059;MWSmce498;mws1434	C05625+C12627+C01714	https:\\www.genome.jp\dbget-bin\www_bget?map00944
Diterpenoid biosynthesis	ko00904	1	2	34	99	Cmmn012997	C06087	https:\\www.genome.jp\dbget-bin\www_bget?map00904
Phenylalanine metabolism	ko00360	1	10	34	99	mws0346	C11457	https:\\www.genome.jp\dbget-bin\www_bget?map00360
Histidine metabolism	ko00340	1	4	34	99	mws0982	C05127	https:\\www.genome.jp\dbget-bin\www_bget?map00340
Nicotinate and nicotinamide metabolism	ko00760	1	2	34	99	pme1137	C01020	https:\\www.genome.jp\dbget-bin\www_bget?map00760
ABC transporters	ko02010	1	4	34	99	pme2292	C00134	https:\\www.genome.jp\dbget-bin\www_bget?map02010
Phenylalanine, tyrosine and tryptophan biosynthesis	ko00400	1	2	34	99	pmb1096	C00463	https:\\www.genome.jp\dbget-bin\www_bget?map00400
Benzoxazinoid biosynthesis	ko00402	1	1	34	99	pmb1096	C00463	https:\\www.genome.jp\dbget-bin\www_bget?map00402
Thiamine metabolism	ko00730	1	1	34	99	pmb0764	C04294	https:\\www.genome.jp\dbget-bin\www_bget?map00730
Biosynthesis of cofactors	ko01240	1	6	34	99	pmb0764	C04294	https:\\www.genome.jp\dbget-bin\www_bget?map01240
Polyketide sugar unit biosynthesis	ko00523	1	1	34	99	pmp000509	C11918	https:\\www.genome.jp\dbget-bin\www_bget?map00523
Ubiquinone and other terpenoid-quinone biosynthesis	ko00130	1	5	34	99	pme1439	C00811	https:\\www.genome.jp\dbget-bin\www_bget?map00130
Isoquinoline alkaloid biosynthesis	ko00950	1	3	34	99	pme1439	C00811	https:\\www.genome.jp\dbget-bin\www_bget?map00950
Isoflavonoid biosynthesis	ko00943	1	3	34	99	MWSHY0017	C00509	https:\\www.genome.jp\dbget-bin\www_bget?map00943

Table S2. KEGG pathways and annotated compounds in the comparison group SD9 vs. SD6

Kegg_pathway	ko_ID	Sig_compound	compound	Sig_compound_all	compound_all	IndexList	CIDList	Pathway
Phenylalanine metabolism	ko00360	4	10	28	96	Lmmn001643;Lmbn002648;MWSmce248;MWS4301	C01772+C02763+C12621+C05593	https:\\www.genome.jp\dbget-bin\www_bget?map00360
Biosynthesis of various plant secondary metabolites	ko00999	3	8	28	96	Lmmn001643;pmb0501;Zmpn003044	C01772+C00179+C09315	https:\\www.genome.jp\dbget-bin\www_bget?map00999
Metabolic pathways	ko01100	18	68	28	96	Lmmn001643;pme3083;Lmbn001981;Lmbn002648;Lmlp003161;Lmbn002644;mws0749;MWSmce248;MWS4301;MWSmce548;MA10107783;pmb0501;pme2292;Lmbn002737;pme1439;pme2693;pme2122;Ymjm000148	C01772+C05653+C05585+C02763+C10497+C06758+C00156+C12621+C05593+C00719+C20772+C00179+C00134+C07209+C00811+C02714+C00388+C06046	https:\\www.genome.jp\dbget-bin\www_bget?map01100
Biosynthesis of secondary metabolites	ko01110	12	43	28	96	Lmmn001643;mws0749;MA10107783;pmb0501;Lmsn003297;pme2292;Zmpn003044;pme1439;mws1574;MWSCX015;pmb3074;pme2122	C01772+C00156+C20772+C00179+C16408+C00134+C09315+C00811+C11673+C10945+C12208+C00388	https:\\www.genome.jp\dbget-bin\www_bget?map01110
Isoquinoline alkaloid biosynthesis	ko00950	2	3	28	96	Hmgn001653;pme1439	C16700+C00811	https:\\www.genome.jp\dbget-bin\www_bget?map00950
Tryptophan metabolism	ko00380	1	5	28	96	pme3083	C05653	https:\\www.genome.jp\dbget-bin\www_bget?map00380
Tyrosine metabolism	ko00350	4	11	28	96	Lmbn001981;MWS4301;pme1439;Ymjm000148	C05585+C05593+C00811+C06046	https:\\www.genome.jp\dbget-bin\www_bget?map00350
Arginine and proline metabolism	ko00330	4	7	28	96	Lmlp003161;pmb0501;pme2292;pme2693	C10497+C00179+C00134+C02714	https:\\www.genome.jp\dbget-bin\www_bget?map00330
Ubiquinone and other terpenoid-quinone biosynthesis	ko00130	3	5	28	96	mws0749;MA10107783;pme1439	C00156+C20772+C00811	https:\\www.genome.jp\dbget-bin\www_bget?map00130
Folate biosynthesis	ko00790	1	1	28	96	mws0749	C00156	https:\\www.genome.jp\dbget-bin\www_bget?map00790
Biosynthesis of cofactors	ko01240	2	6	28	96	mws0749;MA10107783	C00156+C20772	https:\\www.genome.jp\dbget-bin\www_bget?map01240
Glycine, serine and threonine metabolism	ko00260	1	1	28	96	MWSmce548	C00719	https:\\www.genome.jp\dbget-bin\www_bget?map00260
ABC transporters	ko02010	2	4	28	96	MWSmce548;pme2292	C00719+C00134	https:\\www.genome.jp\dbget-bin\www_bget?map02010
Flavonoid biosynthesis	ko00941	2	7	28	96	Lmsn003297;pmb3074	C16408+C12208	https:\\www.genome.jp\dbget-bin\www_bget?map00941
D-Amino acid metabolism	ko00470	1	2	28	96	pme2292	C00134	https:\\www.genome.jp\dbget-bin\www_bget?map00470
Glutathione metabolism	ko00480	1	4	28	96	pme2292	C00134	https:\\www.genome.jp\dbget-bin\www_bget?map00480
Tropane, piperidine and pyridine alkaloid biosynthesis	ko00960	1	6	28	96	pme2292	C00134	https:\\www.genome.jp\dbget-bin\www_bget?map00960
Phenylpropanoid biosynthesis	ko00940	5	12	28	96	mws0011;Lhhp120814;pme1439;MWSCX015;pmb3074	C01533+C10453+C00811+C10945+C12208	https:\\www.genome.jp\dbget-bin\www_bget?map00940
Monoterpenoid biosynthesis	ko00902	1	1	28	96	mws1574	C11673	https:\\www.genome.jp\dbget-bin\www_bget?map00902
Stilbenoid, diarylheptanoid and gingerol biosynthesis	ko00945	1	1	28	96	pmb3074	C12208	https:\\www.genome.jp\dbget-bin\www_bget?map00945
Histidine metabolism	ko00340	1	4	28	96	pme2122	C00388	https:\\www.genome.jp\dbget-bin\www_bget?map00340
Flavone and flavonol biosynthesis	ko00944	2	4	28	96	MWSmce498;mws1434	C12627+C01714	https:\\www.genome.jp\dbget-bin\www_bget?map00944
