# Supplementary material for: Traditional Processing Can Enhance the Medicinal Effects of Polygonatum cyrtonema by Inducing Significant Chemical Changes in the Functional Components in Its Rhizomes
Source: Pharmaceuticals (Basel). 2024 Aug 15;17(8):1074. doi: 10.3390/ph17081074 (PMC11359098; doi:10.3390/ph17081074)
Supplement: Supplementary file 1 [file pharmaceuticals-17-01074-s001.zip › Table S3-Potential toxic alkaloids .pdf]

Table S3. Potential toxic alkaloids

Index	Compounds	Formula	Level	Class I	Class II	CPa	CPb	CPc	SD3a	SD3b	SD3c	SD6a	SD6b	SD6c	SD9a	SD9b	SD9c
pma6298	3-Hydroxypyridine	C5H5NO	3	Alkaloids	Pyridine alkaloids	0.00E+00	0.00E+00	0.00E+00	4.35E+05	6.74E+05	4.07E+05	8.10E+05	9.35E+05	9.03E+05	1.20E+06	9.23E+05	1.42E+06
MWSmce249	Chelidamic acid	C7H5NO5	1	Alkaloids	Pyridine alkaloids	3.59E+04	2.95E+04	1.32E+04	7.61E+05	6.51E+05	6.91E+05	1.66E+06	1.47E+06	1.13E+06	1.58E+06	9.20E+05	1.58E+06
pmb0782	Piperidine	C5H11N	3	Alkaloids	Piperidine alkaloids	1.33E+07	1.51E+07	1.26E+07	1.28E+07	1.36E+07	1.39E+07	7.95E+06	8.44E+06	9.25E+06	5.43E+06	5.85E+06	6.20E+06
pmp001198	6-Deoxyfagomine	C6H13NO2	1	Alkaloids	Piperidine alkaloids	2.02E+07	2.18E+07	1.86E+07	1.89E+07	1.85E+07	2.09E+07	1.21E+07	1.36E+07	1.39E+07	8.94E+06	8.95E+06	9.68E+06
Zblp001009	3-pyridine-methanol-O-β-D-glucopyranosyl	C12H17NO6	2	Alkaloids	Pyridine alkaloids	4.37E+05	4.99E+05	6.51E+05	4.84E+05	5.21E+05	5.35E+05	1.51E+06	1.65E+06	1.20E+06	1.80E+06	1.80E+06	1.72E+06
Ysjp000315	Pipecolic acid	C6H11NO2	2	Alkaloids	Piperidine alkaloids	1.55E+07	1.54E+07	1.63E+07	3.84E+07	2.19E+07	3.88E+07	4.44E+07	4.33E+07	3.22E+07	3.82E+07	3.98E+07	4.13E+07
Wayp004564	4-hydroxy-4-(3-pyridyl)-butanoic acid	C9H11NO3	2	Alkaloids	Pyridine alkaloids	0.00E+00	0.00E+00	0.00E+00	4.23E+05	6.60E+05	3.74E+05	4.98E+05	4.46E+05	6.85E+05	4.77E+05	3.11E+05	4.86E+05
Qmgp102003	O-Acetyljervine	C29H41NO4	1	Alkaloids	Steroid alkaloids	3.81E+04	3.06E+04	5.06E+04	2.75E+05	2.72E+05	3.02E+05	1.04E+06	7.66E+05	7.54E+05	8.71E+05	7.09E+05	8.03E+05
pme2268	Trigonelline	C7H7NO2	1	Alkaloids	Pyridine alkaloids	5.60E+06	1.44E+07	1.42E+07	7.12E+06	8.80E+06	6.01E+06	9.62E+06	9.77E+06	1.06E+07	1.42E+07	1.10E+07	1.32E+07
MWSmce460	2-Piperidone	C5H9NO	2	Alkaloids	Piperidine alkaloids	0.00E+00	0.00E+00	0.00E+00	3.72E+05	2.92E+05	4.67E+05	3.99E+05	4.09E+05	3.72E+05	3.96E+05	3.95E+05	3.92E+05
pme1137	6-Hydroxynicotinic acid	C6H5NO3	2	Alkaloids	Pyridine alkaloids	0.00E+00	0.00E+00	0.00E+00	4.61E+06	4.99E+06	4.76E+06	1.45E+07	1.46E+07	9.66E+06	1.51E+07	1.39E+07	1.80E+07
pme1738	3-Carbamyl-1-methylpyridinium;(1-Methylnicotinamide)	C7H9N2O	3	Alkaloids	Pyridine alkaloids	5.17E+06	1.36E+07	1.32E+07	6.58E+06	8.49E+06	5.72E+06	9.47E+06	9.25E+06	9.66E+06	1.38E+07	1.02E+07	1.25E+07
MWStz034	N-Methylsedridine	C9H19NO	3	Alkaloids	Piperidine alkaloids	1.46E+05	1.27E+05	2.01E+05	1.79E+05	1.94E+05	1.98E+05	1.37E+05	1.47E+05	1.58E+05	1.41E+05	1.43E+05	1.52E+05
MWSslk051	Scopine	C8H13NO2	3	Alkaloids	Tropan alkaloids	7.11E+04	8.20E+04	8.91E+04	1.25E+05	1.20E+05	1.29E+05	7.28E+04	1.00E+05	9.48E+04	9.90E+04	9.30E+04	1.10E+05
Salp002057	neoverataline A	C27H41NO8	3	Alkaloids	Steroid alkaloids	0.00E+00	0.00E+00	0.00E+00	0.00E+00	0.00E+00	0.00E+00	1.77E+07	2.07E+07	1.38E+07	2.33E+07	2.92E+07	2.03E+07
Zmmp004274	Tigloidine	C13H21NO2	3	Alkaloids	Tropan alkaloids	2.84E+03	4.96E+03	3.00E+03	6.54E+03	2.07E+04	2.39E+04	1.34E+05	1.28E+05	1.63E+05	3.19E+05	4.17E+05	4.15E+05
Smbp002161	8-ethylnorlobelol	C9H19NO	2	Alkaloids	Piperidine alkaloids	0.00E+00	0.00E+00	0.00E+00	6.73E+04	4.64E+04	6.68E+04	9.30E+04	1.11E+05	1.19E+05	1.77E+05	1.37E+05	1.39E+05
pmn001692	3-O-Acetylhamayne	C18H19NO5	1	Alkaloids	Isoquinoline alkaloids	3.68E+03	2.02E+04	6.32E+03	1.01E+04	8.21E+03	3.66E+03	2.76E+03	7.30E+03	8.94E+03	0.00E+00	0.00E+00	0.00E+00
MWSmce338	N-Hydroxypipecolic acid	C6H11NO3	2	Alkaloids	Piperidine alkaloids	8.69E+03	3.44E+03	2.95E+03	1.81E+05	1.66E+05	1.79E+05	1.55E+05	1.08E+05	1.63E+05	6.64E+04	5.30E+04	5.63E+04
Wdhp006324	(2e)-3-(4-hydroxyphenyl)-n-[2-(4-hydroxyphenyl)ethyl]prop-2-enimidic acid	C17H17NO3	1	Alkaloids	Isoquinoline alkaloids	1.85E+05	5.68E+05	3.94E+05	1.33E+06	1.92E+06	1.77E+06	9.38E+05	1.28E+06	1.49E+06	1.19E+06	1.59E+06	9.91E+05
