# Supplementary material for: Traditional Processing Can Enhance the Medicinal Effects of Polygonatum cyrtonema by Inducing Significant Chemical Changes in the Functional Components in Its Rhizomes
Source: Pharmaceuticals (Basel). 2024 Aug 15;17(8):1074. doi: 10.3390/ph17081074 (PMC11359098; doi:10.3390/ph17081074)
Supplement: Supplementary file 1 [file pharmaceuticals-17-01074-s001.zip › Table S4-Homoisoflavonoids identified in this study.pdf]

Table S4. Homoisoflavonoids identified in this study

Index	Compounds	Formula	Level	CPa	CPb	CPc	SD3a	SD3b	SD3c	SD6a	SD6b	SD6c	SD9a	SD9b	SD9c
Jmyn006493	(3R)-5,7-Dihydroxy-6,8-dimethyl-3-(4'-hydroxybenzyl)-chromone-4-one	C18H18O5	1	2.18E+05	1.72E+06	2.96E+06	1.08E+06	8.34E+05	7.83E+05	6.07E+05	2.44E+06	1.43E+06	2.99E+06	2.18E+06	1.58E+06
Zjmp102041	5,7-dihydroxy-6,8-dimethyl-3-(4'-hydroxy-3'-methoxybenzyl)chroman-4-one	C19H20O6	1	2.57E+05	7.95E+04	2.73E+04	8.42E+05	8.31E+04	6.25E+04	3.70E+04	1.99E+05	1.01E+05	1.37E+05	3.61E+04	6.92E+04
Zjhp090622	(3R)-5,7-dihydroxy-6,8-dimethyl-3-(2'-hydroxy-4'-methoxybenzyl)-chroman-4-one	C19H20O6	1	2.47E+05	8.68E+04	2.37E+04	9.07E+05	6.66E+04	5.84E+04	3.06E+04	2.12E+05	9.18E+04	1.43E+05	4.00E+04	7.16E+04
Hmhp006830	6,8-Dihydroxy-2-(2-hydroxy-4-methoxybenzyl)-7-methyl-3,4-dihydronaphthalen-1(2H)-one	C18H18O6	2	1.30E+06	7.75E+05	1.74E+05	7.67E+05	5.84E+04	3.71E+04	1.21E+04	2.94E+04	1.21E+04	0.00E+00	0.00E+00	0.00E+00
Zjhp090635	5,7-dihydroxy-6,8-dimethyl-3-(4'-methoxybenzyl)-chroman-4-one	C19H20O5	1	1.68E+04	6.05E+04	3.17E+04	5.97E+04	6.03E+04	3.71E+04	2.90E+04	5.14E+04	3.92E+04	7.05E+04	1.71E+04	5.03E+04
Zjmp102042	iso-5,7-dihydroxy-6,8-dimethyl-3-(4'-hydroxy-3'-methoxybenzyl)chroman-4-one	C19H20O6	1	8.01E+05	5.91E+05	1.08E+05	1.11E+06	2.15E+05	2.16E+05	5.18E+04	3.34E+05	1.56E+05	1.13E+05	4.01E+04	7.16E+04
Zjhp090618	5,7-Dihydroxy-6,8-dimethyl-3-(2',4'-hydroxybenzyl)chroman-4-One	C18H18O6	2	1.23E+06	6.85E+05	1.68E+05	6.49E+05	3.12E+04	5.18E+04	5.50E+03	2.02E+04	1.18E+04	3.67E+03	1.01E+05	4.03E+03
Jmyp005575	5,7-Dihydroxy-6,8-dimethyl-3-(3'-hydroxy-4'-methoxybenzyl)-chroma4-one	C19H20O6	1	2.83E+05	8.28E+04	3.57E+04	8.23E+05	6.98E+04	5.43E+04	3.74E+04	2.21E+05	1.08E+05	1.47E+05	4.08E+04	8.13E+04
Zjhp090621	5,7-dihydroxy-6,8-dimethyl-3-(2'-methoxy-4'-hydroxybenzyl)-chroman-4-one	C19H20O6	1	2.73E+05	7.73E+04	3.49E+04	8.28E+05	6.50E+04	4.38E+04	4.21E+04	2.22E+05	9.41E+04	1.53E+05	5.18E+04	6.71E+04
Hmhp006887	6,8-Dihydroxy-2-(4-hydroxybenzyl)-5,7-dimethyl-3,4-dihydronaphthalen-1(2H)-one	C18H18O5	1	2.44E+04	3.17E+05	4.31E+05	1.54E+05	1.27E+05	1.10E+05	7.64E+04	3.41E+05	1.84E+05	3.90E+05	2.55E+05	1.91E+05
Hmdp001950	2',7-Dihydroxy-3',4'-dimethoxyisoflavan	C17H18O5	2	0.00E+00	0.00E+00	0.00E+00	1.98E+07	1.09E+07	1.85E+07	2.18E+07	2.64E+07	1.80E+07	1.74E+07	2.34E+07	2.47E+07
Zjhp090620	5,7-dihydroxy-6,8-dimethyl-3(R,S)-(3'-hydroxy-4'-methoxybenzyl)-chroman-4-one	C19H20O6	1	2.56E+05	7.25E+04	3.71E+04	7.72E+05	7.44E+04	6.67E+04	4.97E+04	2.07E+05	1.13E+05	1.32E+05	4.38E+04	7.19E+04
Zjhp090609	(E)-5,7-dihydroxy-6,8-dimethyl-3-(4'-hydroxybenzylidene)-chroman-4-one	C18H16O5	1	1.54E+04	1.36E+04	3.28E+04	2.18E+04	1.34E+04	1.02E+04	7.34E+03	5.13E+04	1.08E+04	1.30E+04	1.05E+05	1.41E+04
Lamp006064	3-[(3,4-dihydroxyphenyl)methylidene]-5,7-dihydroxy-6-methoxy-2h-1-benzopyran-4-one glucosyl rhamnoside	C29H34O16	1	0.00E+00	0.00E+00	0.00E+00	1.52E+06	1.33E+06	3.37E+06	6.19E+04	3.93E+05	4.22E+05	6.19E+04	5.66E+04	1.48E+04
MWSHY0072	Methylophiopogonanone B	C19H20O5	1	1.77E+04	6.72E+04	3.78E+04	6.31E+04	4.73E+04	2.93E+04	2.65E+04	6.48E+04	4.36E+04	7.56E+04	2.05E+04	4.26E+04
Zjmp102002	ophiopogonanone B	C18H18O5	1	3.67E+04	2.52E+05	4.44E+05	1.82E+05	1.07E+05	1.06E+05	6.71E+04	2.93E+05	1.78E+05	3.86E+05	2.43E+05	1.95E+05
