# Supplementary figures and images for: Traditional Processing Can Enhance the Medicinal Effects of Polygonatum cyrtonema by Inducing Significant Chemical Changes in the Functional Components in Its Rhizomes
Source: Pharmaceuticals (Basel). 2024 Aug 15;17(8):1074. doi: 10.3390/ph17081074 (PMC11359098; doi:10.3390/ph17081074)

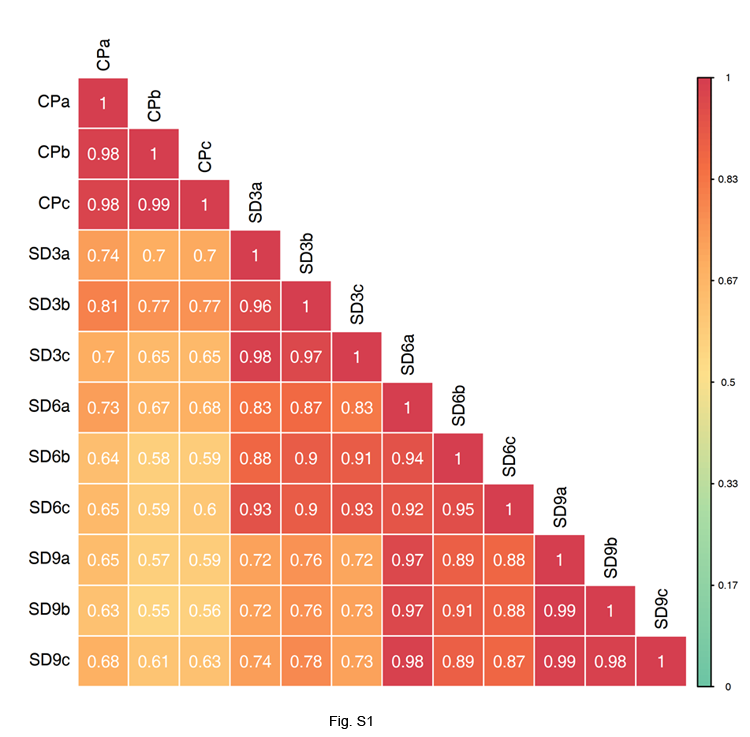

Supplement: Supplementary file 1 [file pharmaceuticals-17-01074-s001.zip › Fig. S1 Correlation analysis of secondary metabolites.tif]
